# A practical guide to the monitoring and management of the complications of systemic corticosteroid therapy

**DOI:** 10.1186/1710-1492-9-30

**Published:** 2013-08-15

**Authors:** Dora Liu, Alexandra Ahmet, Leanne Ward, Preetha Krishnamoorthy, Efrem D Mandelcorn, Richard Leigh, Jacques P Brown, Albert Cohen, Harold Kim

**Affiliations:** 1 Division of Endocrinology and Metabolism, University of Ottawa, The Ottawa Hospital, Ottawa, ON, Canada; 2 University of Ottawa, Children’s Hospital of Eastern Ontario, Ottawa, ON, Canada; 3 McGill University, Montreal Children’s Hospital, Montreal, QC, Canada; 4 Department of Ophthalmology and Vision Sciences, University of Toronto, University Health Network, Toronto Western Hospital, Toronto, ON, Canada; 5 Division of Respiratory Medicine, University of Calgary, Calgary, AB, Canada; 6 Department of Medicine, Laval University, Quebec City, QC, Canada; 7 McGill University, Jewish General Hospital, Montreal, QC, Canada; 8 Western University, London, ON, Canada; 9 McMaster University, Hamilton, ON, Canada; 10 525 Belmont Ave West, Suite 205, Kitchener, ON N2M 5E2, Canada

**Keywords:** Adverse events, Adrenal suppression, Corticosteroids, Cushing’s syndrome, Hyperglycemia, Glaucoma, Glucocorticoids, Glucocorticoid-induced osteoporosis, Side effects, Systemic

## Abstract

Systemic corticosteroids play an integral role in the management of many inflammatory and immunologic conditions, but these agents are also associated with serious risks. Osteoporosis, adrenal suppression, hyperglycemia, dyslipidemia, cardiovascular disease, Cushing’s syndrome, psychiatric disturbances and immunosuppression are among the more serious side effects noted with systemic corticosteroid therapy, particularly when used at high doses for prolonged periods. This comprehensive article reviews these adverse events and provides practical recommendations for their prevention and management based on both current literature and the clinical experience of the authors.

## Introduction

Since their discovery in the 1940s, corticosteroids have become one of the most widely used and effective treatments for various inflammatory and autoimmune disorders (see Table 1). They are used as replacement therapy in adrenal insufficiency (at physiologic doses) as well as in supraphysiologic doses for the management of various dermatologic, ophthalmologic, rheumatologic, pulmonary, hematologic, and gastrointestinal (GI) disorders. In the field of respirology, systemic corticosteroids are used for the treatment of acute exacerbations of chronic obstructive pulmonary disease (COPD) and severe, uncontrolled asthma, as well as for inflammatory parenchymal lung diseases such as hypersensitivity pneumonitis and immune-mediated vasculitis. These are just some of the many important uses of this group of medications that are utilized in almost all areas of medicine.

**Table 1:** Common clinical uses of systemic corticosteroids

**Field of medicine**	**Disorder(s)**
Allergy and respirology	• Moderate to severe asthma exacerbations
	• Acute exacerbations of chronic obstructive pulmonary disease
	• Allergic rhinitis
	• Atopic dermatitis
	• Urticaria/angioedema
	• Anaphylaxis
	• Food and drug allergies
	• Nasal polyps
	• Hypersensitivity pneumonitis
	• Sarcoidosis
	• Acute and chronic eosinophilic pneumonia
	• Interstitial lung disease
Dermatology	• Pemphigus vulgaris
	• Acute, severe contact dermatitis
Endocrinology*	• Adrenal insufficiency
	• Congenital adrenal hyperplasia
Gastroenterology	• Ulcerative colitis
	• Crohn’s disease
	• Autoimmune hepatitis
Hematology	• Lymphoma/leukemia
	• Hemolytic anemia
	• Idiopathic thrombocytopenic purpura
Rheumatology/immunology	• Rheumatoid arthritis
	• Systemic lupus erythematosus
	• Polymyalgia rheumatica
	• Polymyositis/dermatomyositis
	• Polyarteritis
	• Vasculitis
Ophthalmology	• Uveitis
	• Keratoconjunctivitis
Other	• Multiple sclerosis
	• Organ transplantation
	• Nephrotic syndrome
	• Chronic active hepatitis
	• Cerebral edema

NOTE: Systemic corticosteroid uses are not limited to those listed in this table. These agents can be used in almost all areas of medicine.

*In endocrinology, corticosteroid doses are often given at or close to physiologic doses rather than in therapeutic ranges.

Despite their beneficial effects, long-term systemic (oral or parenteral) use of these agents is associated with well-known adverse events (AEs) including: osteoporosis and fractures; adrenal suppression (AS); hyperglycemia and diabetes; cardiovascular disease (CVD) and dyslipidemia, dermatological and GI events; psychiatric disturbances; and immunosuppression. The objectives of this article are to: briefly review the properties and mechanisms of action of systemic corticosteroids; discuss the AEs most commonly associated with long-term use of these agents; and provide practical recommendations for patient monitoring and the prevention and management of these AEs.

## Properties and mechanisms of action of corticosteroids

Corticosteroids are synthetic analogues of the natural steroid hormones produced by the adrenal cortex. Like the natural hormones, these synthetic compounds have glucocorticoid (GC) and/or mineralocorticoid properties. Mineralocorticoids affect ion transport in the epithelial cells of the renal tubules and are primarily involved in the regulation of electrolyte and water balance. GCs, on the other hand, are predominantly involved in carbohydrate, fat and protein metabolism, and have anti-inflammatory, immunosuppressive, anti-proliferative, and vasoconstrictive effects (Table 2) [1].

**Table 2:** **Primary effects of glucocorticoids (GCs) **[1]

	
**Anti-inflammatory:**	Inhibit inflammation by blocking the action of inflammatory mediators (transrepression), or by inducing anti-inflammatory mediators (transactivation)
**Immunosuppressive:**	Suppress delayed hypersensitivity reactions by directly affecting T-lymphocytes
**Anti-proliferative:**	Inhibition of DNA synthesis and epidermal cell turnover
**Vasoconstrictive:**	Inhibit the action of histamine and other vasoconstrictive mediators

*DNA* deoxyribonucleic acid.

Most of the anti-inflammatory and immunosuppressive actions of GCs are attributable, either directly or indirectly, to their interaction with the cytosolic GC receptor, which alters gene transcription to either induce (transactivate) or repress (transrepress) gene transcription in both inflammatory leukocytes and in structural cells, such as epithelium [2-4]. Thus, GCs exert their clinical effects predominantly by upregulating the transcription of anti-inflammatory genes (transactivation) or by downregulating the transcription of inflammatory genes (transrepression) to affect the downstream production of a number of pro-inflammatory cytokine and chemokine proteins, cell adhesion molecules and other key enzymes involved in the initiation and/or maintenance of the host inflammatory response [3,5-7].

## Systemic corticosteroids available in Canada

A number of systemic corticosteroid compounds are commercially available in Canada. These agents differ with respect to potency, duration of action and ratio of mineralocorticoid to GC properties, which determine the corticosteroid’s efficacy and therapeutic use (see Table 3) [1,8].

**Table 3:** Properties, dosing equivalents and therapeutic indications of systemic corticosteroids, relative to hydrocortisone

	Approximate equivalent dose* (mg)	**Relative glucocorticoid activity**	**Relative mineralocorticoid activity**	**Duration of action (hours)**	**General therapeutic indications**
**Glucocorticoids**					
*Short-acting*					
Hydrocortisone	20	1	1	8-12	• Relatively high mineralocorticoid activity makes it suitable for use in adrenal insufficiency
Cortisone	25	0.8	0.8	8-12	• Similar to hydrocortisone
*Intermediate-acting*					
Prednisone	5	4	0.8	12-36	• High glucocorticoid activity makes it useful for long-term treatment, and as an anti-inflammatory/ immunosuppressant
Prednisolone	5	4	0.8	12-36	• Similar to prednisone
Methylprednisolone	4	5	Minimal	12-36	• Anti-inflammatory/immunosuppressant
Triamcinolone	4	5	0	12-36	• Anti-inflammatory/immunosuppressant
*Long-acting*					
Dexamethasone	0.75	30	Minimal	36-72	• Anti-inflammatory/immunosuppressant; used especially when water retention is undesirable given its minimal mineralocorticoid activity
					• Usually reserved for short-term use in severe, acute conditions given its high potency and long-duration of action
Betamethasone	0.6	30	Negligible	36-72	• Similar to dexamethasone
**Mineralocorticoids**					
Fludrocortisone	**	10-15	125-150	12-36	• Used for aldosterone replacement

Table adapted from NICE, 2012 [1]; Furst et al., 2012 [8].

*Equivalent dose shown is for oral or IV administration. Relative potency for intra-ocular or intramuscular administration may vary considerably.

**Glucocorticoid doses which provide a mineralocorticoid effect that is approximately equivalent to 0.1 mg of fludrocortisone are: prednisone or prednisolone 50 mg, or hydrocortisone 20 mg.

Prednisone is perhaps the most widely used of the systemic corticosteroids. Given its high GC activity relative to mineralocorticoid activity, it is generally used as an anti-inflammatory and immunosuppressive agent. Although similar to prednisone and prednisolone, methylprednisolone has even less mineralocorticoid activity and, therefore, may be preferred when mineralocorticoid effects (e.g., water retention) are particularly undesirable [9]. Dexamethasone also has minimal mineralocorticoid activity, but it is much more potent and has a longer duration of action than prednisone and prednisolone. Given its high potency, long-term treatment with dexamethasone is associated with severe hypothalamic-pituitary-adrenal (HPA) axis suppression; therefore, it is generally reserved for short-term use in very severe, acute conditions. Also, its long duration of action makes it unsuitable for alternate-day therapy [9].

Cortisone and hydrocortisone are the least potent GCs. Because these agents have both mineralocorticoid and GC activity, they are generally preferred for use in patients with adrenal insufficiency. Fludrocortisone has much greater mineralocorticoid vs. GC potency and, therefore, is commonly used to replace aldosterone in Addison's disease and the classic salt-wasting form of congenital adrenal hyperplasia [1,8].

## Corticosteroid dosing and relationship to adverse events

A thorough review of corticosteroid dosing is beyond the scope of this manuscript since dosages must be individualized based on the pharmacokinetics of the different preparations, the underlying condition being treated, potential drug interactions with concurrently administered non-steroid agents, and patient response to GC treatment. In non-endocrine disorders, GCs are commonly given in pharmacologic (therapeutic) doses to suppress inflammation. In endocrine disorders, however, corticosteroid doses are often given at or close to physiologic doses (rather than in therapeutic ranges).

GC-associated toxicity appears to be related to both the average dose and cumulative duration of GC use. However, for most GC-related AEs, a “threshold” dose or treatment duration has not been established [10]. The following section provides a comprehensive review of the most common AEs associated with long-term systemic corticosteroid use.

## Adverse events associated with long-term systemic corticosteroid use

### Adults

The most common GC-associated AEs noted in adults include: osteoporosis and fractures; HPA-axis suppression; Cushingoid appearance and weight gain; hyperglycemia/diabetes; CVD and dyslipidemia; myopathy; cataracts and glaucoma; psychiatric disturbances; immunosuppression; as well as other GI and dermatologic events.

#### Osteoporosis, fractures and osteonecrosis

GCs have been shown to stimulate osteoclastic activity initially (first 6–12 months of therapy), followed by a decrease in bone formation by suppressing osteoblastic activity in the bone marrow, decreasing osteoblast function and life span, and promoting the apoptosis of osteoblasts and osteocytes [11-13]. A meta-analysis of over 80 studies in adults found that use of ≥5 mg/day of prednisolone (or equivalent) was associated with significant reductions in bone mineral density (BMD) and an increase in fracture risk within 3 to 6 months of treatment initiation; this increased fracture risk was independent of patient age, gender and the underlying disease [14]. Kanis and colleagues examined 42,500 subjects from seven prospectively studied cohorts followed for 176,000 patient-years and found that prior and current use of corticosteroids increased fracture risk in both adult men and women, regardless of BMD and prior fracture history [15].

Osteonecrosis develops in 9–40% of adult patients receiving long-term GC therapy; it can occur as a result of systemic therapy or via intra-articular injections as well as in the absence of GC-induced osteoporosis [16]. Osteonecrosis is also being increasingly reported in children and adolescents treated for acute lymphoblastic leukemia (ALL) and non-Hodgkin lymphoma [17,18].

Although the risk of osteonecrosis appears to increase with higher doses and prolonged treatment, it may also occur with low doses or after short-term GC exposure. Excessive alcohol intake, hypercoaguable states, sickle cell disease, radiation exposure and human immunodeficiency virus (HIV) infection have also been associated with the development of osteonecrosis [19].

A study of 270 adult cases of GC-induced osteonecrosis of the femoral head indicated that this condition was often misdiagnosed as lumbar disorders [20]. In this study, only one patient did not report any pain associated with osteonecrosis. Of the 269 patients who did report symptoms, 79% experienced pain due to osteonecrosis within 3 years of GC initiation (median 18 months) [20].

#### Adrenal suppression

Adrenal suppression (AS) refers to decreased or inadequate cortisol production that results from exposure of the HPA axis to exogenous GCs [21]. Duration of GC therapy and doses of GC treatment are not reliable predictors of which patients will develop AS [22,23]. AS has been demonstrated after exposure to even 5-days’ duration of high-dose GC therapy [23]. It is important to recognize that inhaled, topical and intraocular GCs may also be absorbed systemically to the degree that they can cause AS [24-26].

Longer-acting GC formulations tend to be associated with a higher risk of AS [27]. Timing of GC administration may also influence the development of AS, with morning administration being potentially less suppressive than evening doses [27,28]. Alternate-day therapy is also theoretically less suppressive than daily GCs based on the physiology of the HPA axis; however, there is currently no solid clinical evidence to support this proposition.

The physiologic effects of cortisol are wide-ranging and are particularly important during times of physiologic stress (i.e., illness or surgery). The clinical presentation of AS is variable; many of the signs and symptoms are non-specific and can be mistaken for symptoms of intercurrent illness or the underlying condition being treated with GC therapy (see Table 4).

**Table 4:** Signs and symptoms of AS and adrenal crisis

	
**Adrenal suppression:**	• Weakness/fatigue
	• Malaise
	• Nausea
	• Vomiting
	• Diarrhea
	• Abdominal pain
	• Headache (usually in the morning)
	• Fever
	• Anorexia/weight loss
	• Myalgia
	• Arthralgia
	• Psychiatric symptoms
	• Poor linear growth in children
	• Poor weight gain in children
**Adrenal crisis:**	• Hypotension
	• Decreased consciousness
	• Lethargy
	• Unexplained hypoglycemia
	• Hyponatremia
	• Seizure
	• Coma

AS often occurs following abrupt discontinuation of GC therapy [29]. However, there are currently no evidence-based guidelines for tapering of GCs. Gradual GC tapering is frequently part of treatment protocols to reduce the risk of relapse and, therefore, comparative studies looking at AS without tapering would be difficult to perform. A study of patients with rheumatic disease found that rapidity of steroid taper did not make a difference in HPA-axis recovery [30]. However, many of these patients had undergone a gradual taper to prevent disease relapse and were all on “close to” physiologic doses of GC at the time of testing. In a study of children with ALL, GC tapering before discontinuation did not lead to complete resolution of AS [31]. Despite the lack of supportive evidence, many centres follow empiric tapering regimes based on the knowledge that AS is often seen following abrupt GC withdrawal.

#### Cushingoid appearance and weight gain

Prolonged corticosteroid therapy commonly causes weight gain and redistribution of adipose tissue that result in Cushingoid features (truncal obesity, facial adipose tissue [i.e., moon face], and dorsocervical adipose tissue). A survey of 2,167 long-term GC users (mean prednisone equivalent dose = 16 ± 14 mg/day for ≥60 days) found weight gain to be the most common self-reported AE (70%) [32]. An analysis of four prospective trials of GC use in patients with rheumatoid arthritis found a 4 to 8% increase in mean body weight with the use of 5–10 mg/day of prednisone or equivalent for >2 years [10].

Cushingoid features may develop within the first two months of GC therapy, and the risk of these complications appears to be dependent on both the dose and duration of treatment. One study evaluating the prevalence of Cushingoid abnormalities in 88 patients initiating long-term systemic corticosteroid therapy (initial daily dose ≥20 mg of prednisone or equivalent) found the cumulative incidence rates of these abnormalities to be 61% at 3 months and almost 70% at 12 months [33]. The risk of these complications was higher in younger patients, those with a higher baseline body mass index (BMI) and those with a higher initial caloric intake (>30 kcal/kg/day). Another study found the rate of Cushingoid features to increase linearly with dose: 4.3%, 15.8%, and 24.6% in patients receiving <5 mg/day, 5–7.5 mg/day, and >7.5 mg/day of prednisone (or equivalent), respectively [34].

#### Hyperglycemia and diabetes

Exogenous corticosteroid use is associated with hyperglycemia, and high-dose therapy increases insulin resistance in patients with pre-existing and new-onset diabetes. The effects of GC administration on glucose levels are observed within hours of steroid exposure [35], and appear to be dose-dependent. A population-based study of over 11,000 patients found that the risk for hyperglycemia increased substantially with increasing daily steroid dose; odds ratios (ORs) for hyperglycemia were 1.77, 3.02, 5.82 and 10.34 for 1–39 mg/day, 40–79 mg/day, 80–119 mg/day and ≥120 mg/day of hydrocortisone-equivalent, respectively [36]. GCs also appear to have a greater impact on postprandial compared to fasting glucose levels [37].

Glycemic targets and management strategies for patients with GC-induced hyperglycemia/diabetes are generally the same as in those with pre-established diabetes or glucose intolerance in the absence of GC therapy [38,39] (see *Hyperglycemia/Diabetes* sections in *Practical Recommendations for the Monitoring, Prevention and Management of Systemic Corticosteroid-Induced AEs*). In general, GC-induced hyperglycemia improves with dose reductions and usually reverses when steroid therapy is discontinued, although some patients may develop persistent diabetes.

#### Cataracts and glaucoma

The risk of both cataracts and glaucoma is increased in patients using GCs, and this risk appears to be dose-dependent. GC use is typically associated with the development of posterior subcapsular cataracts (PSCC) [40], as opposed to nuclear or cortical cataracts. PSCC tend to be more visually significant and, therefore, usually require earlier surgical intervention/removal than other types of cataracts.

There is inter-individual variation in susceptibility to PSCC and the incidence varies per individual. Time until onset is at least 1 year with doses ≥10 mg/day of oral prednisone (or equivalent). Although PSCC are frequently seen in patients treated systemically, or even occasionally in those receiving inhaled corticosteroids (ICSs) [41], they are more commonly caused secondary to local treatment (e.g., topical eye drops and periocular or intravitreal administration).

Glaucoma is the more serious ocular complication of GC therapy. Systemic corticosteroids can painlessly increase intraocular pressure, leading to visual field loss, optic disc cupping, and optic nerve atrophy. Once systemic therapy is discontinued, the elevation in intraocular pressure often resolves within a few weeks, but the resultant damage to the optic nerve is often permanent.

While all patients using systemic steroids are at risk for elevation in intraocular pressure and glaucoma, certain groups appear to be at higher risk. Ocular hypertension and glaucomatous visual field defects have been reported in patients using systemic steroids with a personal or family history of open angle glaucoma, diabetes, high myopia or connective tissue disease (particularly rheumatoid arthritis) [42]. To reduce the risk of steroid-induced glaucoma, it is important to screen patients for these risk factors. All patients who may require long-term systemic GC therapy with a positive history for glaucomatous risk factors should be referred to an ophthalmologist for a comprehensive ocular assessment (see *Ophthalmologic Examination* section).

Central serous chorioretinopathy (CSCR) is also associated with systemic GC use. This type of chorioretinopathy is associated with the formation of subretinal fluid in the macular region which leads to separation of the retina from its underlying photoreceptors. This manifests as central visual blur and reduced visual acuity. Therefore, GCs should be used cautiously in patients with a history of CSCR [43].

#### Cutaneous adverse events

Corticosteroids induce atrophic changes in the skin that can lead to skin thinning and fragility, purpura and red striae. Skin thinning and purpura are usually reversible upon discontinuation of therapy, but striae are permanent.

Purpura generally affect the sun exposed areas of the dorsum of the hands and forearms, as well as the sides of the neck, face, and lower legs, and are usually not accompanied by palpable swelling [44,45]. Red striae generally appear on the thighs, buttocks, shoulders and abdomen.

Impairment of wound healing is another common, and potentially serious, side effect of systemic GC use. Corticosteroids interfere with the natural wound-healing process by inhibiting leukocyte and macrophage infiltration, decreasing collagen synthesis and wound maturation, and reducing keratinocyte growth factor expression after skin injury [44]. Some topical and systemic agents may help counter the effects of corticosteroids on wound healing, including epidermal growth factor, transforming growth factor beta, platelet-derived growth factor, and tetrachlorodecaoxygen [45].

#### Gastrointestinal events

GC therapy has been associated with an increased risk of several adverse GI events including gastritis, ulcer formation with perforation and hemorrhage, dyspepsia, abdominal distension and esophageal ulceration. Despite the commonly held perception that steroid use increases the risk of peptic ulcer disease, large meta-analyses of randomized, controlled trials have failed to show a significant association between GC use and peptic ulcers [46,47]. Recent evidence suggests that the risk of peptic ulcer disease due to corticosteroids alone is low, but increases significantly when these agents are used in combination with non-steroidal anti-inflammatory drugs (NSAIDS) [48]. One meta-analysis found a nearly four-fold increased risk of GI events among GC users who were also taking NSAIDS vs. those not using NSAIDS [49]. Messer et al. also found a four-fold increased risk of GI events with concomitant NSAID and GC use vs. non-use of either drug [50].

Acute pancreatitis has also been reported to be an adverse effect of corticosteroid use. A Swedish population-based, case–control study demonstrated an increased risk of acute pancreatitis after exposure to GC therapy [51]. Overall, the OR for developing acute pancreatitis was 1.53 (95% confidence interval [CI], 1.27-1.84) in GC users vs. non-users, with the risk of developing pancreatitis appearing to be greatest 4–14 days after subjects received treatment [51]. However, other evidence suggests that the underlying disease processes for which GC therapy is prescribed (particularly systemic lupus erythematosus [SLE]) may be more likely causes of pancreatitis than GC use [52].

#### Cardiovascular disease and dyslipidemia

GC use is associated with AEs that are known to be associated with a higher CVD risk, including hypertension, hyperglycemia, and obesity. A population-based study comparing 68,781 GC users and 82,202 non-users found the rate of CV events to be significantly higher in patients prescribed high GC doses (≥7.5 mg/day of prednisone or equivalent) vs. those who had not received GCs (adjusted relative risk [RR], 2.56; 95% CI, 2.18-2.99); CV risk was not increased in patients using <7.5 mg of prednisone daily [53]. Another large, retrospective case–control study found current GC use to be associated with a significantly increased risk of heart failure (adjusted OR, 2.66; 95% CI, 2.46-2.87) and ischemic heart disease (OR, 1.20; 95% CI, 1.11-1.29), but not ischemic stroke or transient ischemic attack (TIA). CV risk was found to be greater with higher GC doses and with current vs. past use [54].

Population-based studies conducted in Northern Europe have also noted an increased risk of new-onset atrial fibrillation (AF) and flutter in GC users [55,56]. In these studies, the risk of AF was significantly greater with current or recent use (i.e., within 1 month) of high GC doses or with long-term use of these agents.

Serious CV events, including arrhythmias and sudden death, have also been reported with pulse GC therapy. However, these events are rare and have occurred primarily in patients with underlying kidney or heart disease [57]. Although it is unclear whether these serious AEs are due to GC use or the underlying condition, some experts recommend continuous cardiac monitoring in patients with significant cardiac or kidney disease receiving pulse therapy. Longer infusion times (2–3 h) for pulse GC therapy should be considered in patients who are at high risk of CVD [58].

Findings from studies examining the relationship between GC use and dyslipidemia have been conflicting. While clinical trials involving patients with SLE have shown prednisone doses >10 mg/day to be associated with hyperlipidemia [59,60], another trial conducted in patients with rheumatoid arthritis found no adverse effect of prednisone (20 mg/day tapered to 5 mg/day over 3 months) on serum lipids after adjustment for other risk factors [61]. In fact, findings from a study examining data from 15,004 participants in the Third National Health and Nutrition Examination Survey suggest that GC use may have a beneficial effect on lipids in adults ≥60 years of age [62]. Despite the conflicting evidence, regular monitoring of lipids (as well as other traditional risk factors for CVD) is recommended in patients using GCs at high doses or for prolonged periods (see *CV Risk and Dyslipidemia* section).

#### Myopathy

Corticosteroids have direct catabolic effects on skeletal muscles that can lead to reductions in muscle protein synthesis and protein catabolism and, ultimately, muscle weakness. Myopathy generally develops over several weeks to months of GC use. Patients typically present with proximal muscle weakness and atrophy in both the upper and lower extremities; myalgias and muscle tenderness, however, are not observed. [58,63].

Although there is some variation in the dose and duration of GC treatment prior to the onset of myopathy, it is more common in patients treated with ≥ 10 mg/day of prednisone or equivalent [64]. Also, the higher the GC dose utilized, the more rapid the onset of muscle weakness.

There is no definitive diagnostic test for GC-induced myopathy and, therefore, the diagnosis is one of exclusion. Symptoms generally improve within 3 to 4 weeks of dose reductions, and usually resolve after discontinuation of GC therapy [64]. Evidence also suggests that both resistance and endurance exercise may help attenuate GC-induced muscle atrophy [65].

Critical illness myopathy may also develop in patients requiring large doses of IV GCs and neuromuscular blocking agents. It is characterized by severe, diffuse proximal and distal weakness that develops over several days. Although it is usually reversible, critical illness myopathy can lead to prolonged intensive care unit (ICU) admissions, increased length of hospital stays, severe necrotizing myopathy and increased mortality [58,63,66]. Treatment is directed toward discontinuation of GC therapy or dose reductions as soon as possible, as well as aggressive management of underlying medical comorbidities.

#### Psychiatric and cognitive disturbances

GC use can lead to a wide range of psychiatric and cognitive disturbances, including memory impairment, agitation, anxiety, fear, hypomania, insomnia, irritability, lethargy, mood lability, and even psychosis. These AEs can emerge as early as 1 week after initiating corticosteroid therapy, and appear to be dependent on dose and duration of therapy [67,68]. A family history of depression or alcoholism has also been reported as a risk factor for the development of GC-related affective disorders [69]. Individuals who develop psychiatric manifestations on short courses of GCs most commonly report euphoria, while those on long-term therapy tend to develop depressive symptoms [68,70,71].

GC therapy may also be associated with sleep disturbances and unpleasant dreams [72]; the risk of these events can potentially be decreased by modifying the timing of GC administration (e.g., a single morning dose) and/or night-time administration of drugs with sedative effects.

A decline in declarative and working memory has also been reported with GC therapy; these effects appear to be dose-dependent and frequently occur during the first few weeks of therapy [73]. Partial loss of explicit memory has been reported in patients treated with prednisone doses of 5 to 40 mg/day for at least 1 year [74]. Older patients appear to be more susceptible to memory impairment with less protracted treatment.

GC-induced psychosis usually only occurs with the use of high doses (>20 mg of prednisone or equivalent) for prolonged periods [75]. In patients with SLE, low serum albumin levels may also be predictive of GC-induced psychosis [76]. For patients with persistent symptoms of psychosis, antipsychotic therapy may be required [63].

Most patients with psychiatric reactions to corticosteroids usually recover from these symptoms with dose reductions or upon cessation of therapy. Lithium has also been found to be effective for both the prophylaxis and management of GC-related affective disorders [77].

#### Immunosuppression

The mechanisms by which corticosteroids inhibit the immune system and decrease inflammation may predispose patients to infection. A meta-analysis of 71 clinical trials involving over 2000 patients randomly allocated to systemic GC therapy found the overall rate of infectious complications to be significantly higher in patients using systemic corticosteroids vs. control subjects (RR, 1.6; 95% CI, 1.3-1.9; P < 0.001). However, the rate was not increased in patients given a daily dose of < 10 mg or a cumulative dose of < 700 mg of prednisone [78].

In addition to GC dose, other factors influencing the risk of infection include: the underlying disorder, patient age, and concomitant use of immunosuppressive or biologic therapies [48,79]. A study comparing the infection risk posed by biologic therapies vs. non-biological disease-modifying antirheumatic drugs (DMARDs) in patients with rheumatoid arthritis found baseline GC use to be the factor most strongly associated with serious infections [80].

Patients using GCs appear to be particularly susceptible to invasive fungal and viral infections; this is especially true in bone marrow transplant recipients [45]. Older patients and those with lower functional status are also at higher risk for infections with steroid use. It is important to note that early recognition of infections in patients taking GCs is often difficult [48]. GC users may not manifest signs and symptoms of infection as clearly as non-users, due to the inhibition of cytokine release and associated reduction in inflammatory and febrile responses.

### Children & adolescents

The GC-induced AEs seen in adults can also occur in the pediatric population, including osteoporosis, hyperglycemia, Cushing’s syndrome and AS. However, one side effect that is unique to children is growth suppression.

#### Growth suppression

Oral GC therapy has been associated with a delay in growth and puberty in children with asthma and other childhood diseases such as nephrotic syndrome [81-85]. Some evidence suggests that final height may also be compromised in children with a history of GC use [81,86]. Lai and colleagues reported growth data on 224 children with mild-to-moderate cystic fibrosis who participated in a trial of alternate-day prednisone (1 or 2 mg/kg body weight) vs. placebo [86]. Subjects started prednisone treatment at a mean age of 9.5 years (range, 6 to 14 years); treatment was discontinued at mean ages of 12.9 and 13.8 years for the high-dose and low-dose groups, respectively, and growth was followed for an additional 6 to 7 years after prednisone discontinuation. At the time of final follow-up, 152 patients (68%) were older than 18 years of age. Mean height after age 18 years was found to be significantly lower in boys previously treated with either high- or low-dose prednisone vs. placebo. When indices of pulmonary status were controlled for, the negative association between the use of prednisone and height remained strong in boys. No persistent growth impairment was noted in female subjects.

It is important to note that although growth can be an independent adverse effect of corticosteroid therapy, it can also be a sign of AS.

#### Adrenal suppression

AS is the most common cause of adrenal insufficiency in children. The rate of adrenal crisis or death related to AS is unknown, however, adrenal insufficiency is associated with higher mortality in the pediatric population, highlighting the importance of recognition [29]. As in adults, the symptoms of AS are non-specific; therefore, the condition may go unrecognized until exposure to a physiological stress (illness, surgery or injury), which may result in adrenal crisis. Children with adrenal crisis secondary to AS may present with hypotension, shock, decreased consciousness, lethargy, unexplained hypoglycemia, seizures or even death (see Table 4) [87-91].

Several cases of pediatric AS have been reported in the literature, including adrenal crises in children requiring hospitalization and prolonged ICU stays [92,93]. To our knowledge, there have been no published population studies looking at the frequency of symptomatic AS associated with systemic GCs. However, interim results from a national survey examining AS associated with any form of GC in the Canadian pediatric population over a two-year period have reported 44 cases of symptomatic AS, 6 of which presented as adrenal crisis [90]. A recent meta-analysis of AS in children treated with acute lymphoblastic leukaemia (ALL) found biochemical evidence of AS immediately following GC discontinuation in nearly all 189 patients [93]. AS resolved within a few weeks in most patients, but persisted for up to 34 weeks in others.

Although some studies have suggested that higher doses and longer durations of GC treatment may be risk factors for AS, these findings have not been consistent across trials [30,93-96]. Even relatively low pharmacologic GC doses are significantly higher than physiologic doses, making AS a potential risk. For example, the standard dose of prednisone for the treatment of nephrotic syndrome in children is 2 mg/kg/day. When converted into dose/m^2^, this dose is approximately 20 times the physiologic dose of GCs, highlighting the potential for AS with similar therapies. Currently, the Pediatric Endocrine Society recommends that AS be considered in all children who have received supraphysiological doses of GCs (>8-12 mg/m^2^/day hydrocortisone or equivalent) for greater than 2 weeks [29].

#### Hyperglycemia and diabetes

Most cases of medication-induced diabetes in children are associated with GC use. Steroid-induced hyperglycemia and diabetes have been reported in post-transplant patients, children with ALL, and those undergoing treatment for nephrotic syndrome [97,98]. There have also been reports of diabetic ketoacidosis at presentation in these children [97,98].

There is currently limited data describing risk factors for hyperglycemia and diabetes secondary to GC use in the pediatric population. Although obesity has been described as a potential risk factor, a retrospective Canadian study of children < 18 years of age found that, compared to those with established type 2 diabetes, those with medication-induced diabetes were less likely to be obese, have a positive family history of type 2 diabetes, and have obesity-related comorbidities (e.g., dyslipidemia, hypertension or elevated alanine aminotransferase levels) [97]. Therefore, evaluating for the typical type 2 diabetes risk factors may not be sufficient for identifying children at-risk of developing steroid-induced hyperglycemia or diabetes.

#### Cushing’s syndrome

GC therapy is by far the most common cause of Cushing’s syndrome in children. The clinical presentation in the pediatric population is similar to that in adults, and includes truncal obesity, skin changes and hypertension. In children, however, growth deceleration is also observed [99]. Children who develop features of Cushing’s syndrome as a result of GC therapy are at higher risk of experiencing AS. Therefore, HPA-axis function should be evaluated prior to discontinuing steroid therapy in children with Cushingoid features [88,89].

#### Osteoporosis

A number of studies have reported decreased bone density in children taking oral corticosteroids [100-107]. Van Staa and colleagues examined the medical records of general practitioners in the United Kingdom to estimate the fracture incidence rates in children aged 4–17 years taking oral steroids (n = 37,562) and those taking non-systemic corticosteroids (n = 345,748) [108]. The risk of fracture was increased in children who received four or more courses of oral corticosteroids (adjusted OR, 1.32; 95% CI, 1.03-1.69). Of the various fracture types, the risk of humerus fracture was doubled in these children (adjusted OR, 2.17; 95% CI, 1.01-4.67). Children who stopped taking oral corticosteroids had a comparable risk of fracture to those in the control group [108].

Vertebral fractures are an under-recognized manifestation of osteoporosis in children, in part due to the fact that such fractures are often asymptomatic (even when moderate or severe) [109-112]. Similar to adults, vertebral fractures in GC-treated children are most frequently noted in the mid-thoracic region and at the thoracolumbar junction [109-112]. Recently, it has been shown that in children with GC-treated rheumatic disorders, 7% had prevalent vertebral fractures around the time of GC initiation, and 6% manifested incident vertebral fractures at 1 year [110,111]. Children with rheumatic conditions and incident vertebral fractures at 12 months received twice as much steroid, and had greater increases in BMI and declines in spine BMD Z-scores [111]. These studies have played an important role in furthering our understanding of the osteoporosis burden manifesting as vertebral fractures in steroid-treated children.

## Practical recommendations for the monitoring, prevention and management of systemic corticosteroid-induced adverse events

### Assessment and monitoring

Before initiating long-term systemic corticosteroid therapy, a thorough history and physical examination should be performed to assess for risk factors or pre-existing conditions that may potentially be exacerbated by GC therapy, such as diabetes, dyslipidemia, CVD, GI disorders, affective disorders, or osteoporosis. At a minimum, baseline measures of body weight, height, BMD and blood pressure should be obtained, along with laboratory assessments that include a complete blood count (CBC), blood glucose values, and lipid profile (Table 5). In children, nutritional and pubertal status should also be examined.

**Table 5:** Assessment and monitoring of patients scheduled for long-term systemic corticosteroid therapy

		
**Baseline:**	*Physical:*	*Investigations:*
	• Weight	• CBC
	• Height	• Glucose (FPG, A1C, 2-h OGTT or casual PG)
	• BMI	• Lipids (LDL-C, HDL-C, TC, non-HDL-C, TG, ± apo B)
	• Blood pressure	• BMD
**Subsequent monitoring:**	*Bone health (adults):*	
	• Annual height measurement, and questionnaire for incident fragility fracture	
	• BMD 1-year post GC initiation	
	→ If stable: assess every 2–3 years	
	→ If decreased: assess annually	
	• Lateral spine x-ray in adults ≥65 years to examine for vertebral fractures	
	• Use FRAX to estimate fracture risk	
	→ Available at: http://www.sheffield.ac.uk/FRAX	
	• Consider referral to endocrinologist/rheumatologist if fracture risk is high and/or BMD is decreasing	
	*Bone health (children):*	
	• Consider a baseline spine BMD and lateral spine x-ray in children receiving ≥3 months of GC therapy	
	• Repeat at intervals (typically yearly) if there is persistence of risk factors:	
	→ Ongoing steroid therapy	→ Declines in spine BMD Z-scores or BMC
	→ Low trauma extremity fractures	→ Growth deceleration
	→ Back pain	→ Cushingoid features
	• Referral to a pediatric bone health specialist if there is evidence of bone fragility (low-trauma extremity	
	or vertebral fractures) or declines in BMD Z-scores	
	*HPA-axis functioning (see Table *8*)*	
	*Growth (Children* &*Adolescents):*	
	• Monitor every 6 months and plot on growth curve	
	• If growth velocity inadequate, refer to pediatric endocrinologist for further assessment	
	*Dyslipidemia and CV Risk (adults):*	
	• Assess lipids 1 month after GC initiation, then every 6–12 months	
	• Assess 10-year CV risk using FRS	
	→ Available at: https://www.cvdriskchecksecure.com/FraminghamRiskScore.aspx	
	*Hyperglycemia/Diabetes:*	
	• Screen for classic symptoms at every visit: polyuria, polydipsia, weight loss	
	• Monitor glucose parameters:	
	→ For at least 48 hours after GC initiation [38]	
	→ Then every 3–6 months for first year; annually thereafter	
	• In children, monitor FPG annually	
	→ Annual OGTT if child is obese or has multiple risk factors for diabetes	
	*Ophthalmologic Examination:*	
	• Refer for annual examination by ophthalmologist	
	→ Earlier examination for those with symptoms of cataracts	
	• Early referral for intra-ocular pressure assessment if:	
	→ Personal or family history of open angle glaucoma	→ Diabetes mellitus
	→ Diabetes mellitus	→ High myopia
	→ High myopia	→ Connective tissue disease (particularly rheumatoid arthritis)
	→ Connective tissue disease (particularly rheumatoid arthritis)	

*BMI* body mass index, *BMC* bone mineral content, *BMD* bone mineral density, *CBC* complete blood count, *FPG* fasting plasma glucose, *A1C* glycated hemoglobin, *PG* plasma glucose, *LDL-C* low-density lipoprotein cholesterol, *HDL-C* high-density lipoprotein cholesterol, *TC* total cholesterol, *TG* triglycerides, *apo B* apolipoprotein B, *FRAX* Fracture Risk Assessment Tool, *CV* cardiovascular, *FRS* Framingham Risk Score, *OGTT* oral glucose tolerance test.

Symptoms of and/or exposure to serious infections should also be assessed as corticosteroids are contraindicated in patients with untreated systemic infections. Patients without a history of chicken pox should be advised to avoid close contact with people who have chickenpox or shingles, and to seek urgent medical advice if they are exposed [1]. Concomitant use of other medications should also be assessed before initiating therapy as significant drug interactions have been noted between GCs and several drug classes [1,8] (see Table 6). Females of childbearing age should also be questioned about the possibility of pregnancy. GC use in pregnancy may increase the risk of cleft palate in offspring, although the absolute risk appears to be low [48].

**Table 6:** **Major drug interactions with systemic GCs **[1,8]

**Interacting drug class**	**Effect**	**Recommendation/comment**
**Anticonvulsants** (e.g., carbamazepine, phenobarbital, phenytoin)	• ↓ GC exposure and efficacy; may persist for weeks following discontinuation of anticonvulsant	• Closely monitor outcomes of concomitant use
		• GC dose alterations may be required
**Anticoagulants** (e.g., warfarin)	• May ↑ anticoagulant effects of warfarin and ↑ risk of GI bleeding	• Monitor INR closely
		• Significant alteration in warfarin dose will likely be required within 3–7 days of GC initiation
**Antifungals** (e.g., itraconazole, ketoconazole)	• ↑ GC exposure and toxicity	• Monitor concurrent use for signs of GC overdose (fluid retention, hypertension, hyperglycemia)
		• Dose alteration of methylprednisolone and dexamethasone may be needed (prednisone and prednisolone not affected to a clinically relevant degree by this interaction)
**Antidiabetic agents**	• GC initiation can lead to glucose dysregulation, thereby counteracting the effects of antidiabetic drugs	• ↑ frequency of BG monitoring when initiating GC therapy
		• Adjust antidiabetic therapy based on BG results
**Antibiotics (macrolides)** (e.g., clarithromycin)	• ↑ GC exposure and toxicity	• Monitor concurrent use for signs of GC overdose (fluid retention, hypertension, hyperglycemia)
		• Dose alteration of methylprednisolone and dexamethasone may be needed (prednisone and prednisolone not affected to a clinically relevant degree by this interaction)
**Antivirals** (e.g., atazanavir, indinavir, ritonavir, saquinavir)	• ↑ GC exposure and toxicity	• Monitor concurrent use for signs of GC overdose (fluid retention, hypertension, hyperglycemia)
	• Dexamethasone may ↑ levels of indinavir and saquinavir	• Dose alteration of methylprednisolone and dexamethasone may be needed (prednisone and prednisolone not affected to a clinically relevant degree by this interaction)
		• Monitor antiviral efficacy of indinavir and saquinavir if patient is taking dexamethasone
**Anti-infectives** (e.g., efavirenz, nevirapine, rifampin)	• ↓ GC exposure and efficacy; may persist for weeks following discontinuation of anti-infective	• Closely monitor outcomes, especially in transplant recipients
		• ↑ GC dose accordingly
**Diuretics, potassium wasting** (e.g., furosemide, HCTZ)	• GCs may ↑ kaliuretic effects of these diuretics	• Monitor potassium levels to determine whether alteration of diuretic therapy and/or potassium supplementation is needed
**Live vaccines**	• Immunization with live vaccines while taking immunosuppressive GC doses (40 mg/day of prednisolone [or equivalent] for > 7 days) may increase risk of both generalized and life-threatening infections	• Postpone live vaccines for at least 3 months after high-dose GC therapy is discontinued
**NSAIDS**	• May ↑ risk of GI ulcers when given concomitantly with corticosteroids	• Consider use of PPI if person is at risk of GI ulcers

*GC* glucocorticoid, *INR* international normalized ratio, *BG* blood glucose, *GI* gastrointestinal, *HCTZ* hydrochlorothiazide, *PPI* proton pump inhibitor, *NSAIDS* non-steroidal anti-inflammatory drugs.

The above-mentioned parameters should be monitored regularly. Specific recommendations for the assessment and monitoring of BMD and fracture risk, diabetes, CV risk and dyslipidemia, AS, growth, and ophthalmologic events are provided below.

#### BMD and fracture risk in adults

The authors recommend annual height measurement and questioning for incident fragility fractures in adults receiving GC therapy. Assessment of BMD at baseline and after 1 year of GC therapy in adults who are expected to be on prednisone ≥5 mg/day (or equivalent) for over 3 months is also recommended. If BMD is stable at the 1-year follow-up and fracture risk is low, then subsequent BMD assessments can be performed every 2–3 years (Table 5). However, if bone density has decreased at the initial 1-year follow-up, both BMD and fracture risk should be assessed annually. Guidelines currently recommend using the World Health Organization’s (WHO) Fracture Risk Assessment Tool (FRAX) to estimate fracture risk in order to determine which patients should be started on pharmacologic therapy for fracture prevention [113-117]. However, it is important to note that FRAX does not differentiate between past and present GC use or steroid doses. Experts recommend adjusting FRAX risk according to GC dose [118] (see Table 7). For high-doses (≥7.5 mg/day of prednisolone or equivalent), 10-year hip fracture risk is increased by ~20% and major osteoporotic fracture risk by ~15%, depending on age. For medium doses (2.5-7.5 mg daily), the unadjusted FRAX value can be used, and for low-dose exposure (<2.5 mg daily of prednisolone or equivalent), the probability of a major fracture is decreased by approximately 20%, depending on age.

**Table 7:** **Percentage adjustment of 10-year probabilities of a hip fracture or a major osteoporotic fracture by age according to dose of GCs **[118]

**Dose: Prednisolone equivalent (mg/day)**	**Age (years)**						
	**40**	**50**	**60**	**70**	**80**	**90**	**All ages**
**Hip fracture**							
Low < 2.5	−40	−40	−40	−40	−30	−30	−35
Medium* 2.5–7.5							
High ≥ 7.5	+25	+25	+25	+20	+10	+10	+20
**Major osteoporotic fracture**							
Low < 2.5	−20	−20	−15	−20	−20	−20	−20
Medium* 2.5–7.5							
High ≥ 7.5	+20	+20	+15	+15	+10	+10	+15

Reproduced from Kanis et al. 2011 [118].

*No adjustment.

A lateral spine x-ray is also recommended in adults ≥65 years to assess for vertebral fractures.

#### BMD and fracture risk in children

In adults, a single BMD assessment can help predict the likelihood of fracture due to age-related osteoporosis. In children with GC-induced osteoporosis, however, this relationship is not as evident. Therefore, experts have recommended serial BMD assessments in at-risk children as well as in those displaying evidence of growth failure [119]. Since BMD results need to be carefully interpreted in relation to the child’s gender, age, height, and weight, as well as the underlying disease requiring GC therapy, referral to a specialist for assessment of bone symptomatology and BMD changes is recommended. At the same time, the bone health assessment of a child on chronic GC therapy needs to be extended beyond BMD in order to identify risk factors as well as early manifestations of osteoporosis. As such, bone health monitoring in pediatric chronic GC users includes an evaluation of calcium and vitamin D intake, back pain, physical activity, and disease-related risk factors for attenuated bone mineral accrual and bone loss (such as chronic inflammation and disuse). A spine radiograph should be considered in at-risk children with a prior history of vertebral fractures, back pain, chronic GC exposure (> 3 months), poorly-controlled inflammatory disease, significantly impaired mobility, or reductions in spine BMD Z-scores on serial measurements (Table 5).

#### Osteonecrosis (adults and children)

Because early diagnosis and appropriate intervention can prevent or delay the progression of osteonecrosis and the need for joint replacement, patients using high-dose GC therapy or those treated with GCs for prolonged periods should be evaluated for joint pain and decreased range of motion at each visit [58]. Magnetic resonance imaging should be considered in adult or pediatric patients presenting with these signs or symptoms [16].

#### Adrenal suppression (AS)

Health care providers must be aware of the risk of AS in patients who have received supraphysiological GC doses. The risk of AS is low in patients who have been treated with GC therapy for less than 1 week [120]. However, as is seen following longer courses of GC treatment, AS may result from multiple short courses of high-dose therapy. Based on current evidence, experts recommend that physicians be aware of the risk of AS in patients receiving supraphysiological GC doses for >2 weeks, those who have received multiple courses of oral steroids totaling >3 weeks in the last 6 months, or in patients presenting with symptoms of AS (including growth failure in children) (see Table 8) [91].

**Table 8:** **Screening recommendations for AS **[91]


**When to Screen?**
*• Patient has received systemic corticosteroids for:*
> 2 consecutive weeks or >3 cumulative weeks in the last 6 months
*• Patient has persistent symptoms of AS:*
– Weakness/fatigue, malaise, nausea, vomiting, diarrhea, abdominal pain, headache (usually in the morning), poor weight gain and/or growth in children, myalgia, arthralgia, psychiatric symptoms, hypotension*, hypoglycemia*
**How to Screen?**
*•* Measure early morning cortisol^‡^
– GC dose tapered to physiologic dose prior to test
– No oral GCs the evening and morning prior to the test^†^
– Must be completed by 8:00 am or earlier
– Fasting not required
*•* If morning cortisol is normal but patient has symptoms of AS, perform low-dose ACTH stimulation test^‡^ to confirm diagnosis:
– 1 μg of cosyntropin; cortisol levels taken at 0, 15–20 and 30 minutes**
– Peak cortisol < 500 nmol/L = AS (peak >500 nmol/L is normal)
**When to be Concerned?**
*•* Early morning cortisol < 85 nmol/L = diagnosis of AS
*•* Early morning cortisol < laboratory normal = possible AS; consider endocrinology referral for confirmation of diagnosis

Modified from Ahmet et al., 2011 [91].

*AS* adrenal suppression, *ACTH* adrenocorticotropic hormone, *GCs* glucocorticoids.

*Symptoms of adrenal crisis require emergent management.

^†^Patients must be switched to hydrocortisone for this to apply. If the patient is on a GC with a longer half-life (e.g., dexamethasone), then morning cortisol will remain suppressed due to the medication 24 hours after a dose.

^**^Ideally, GCs should be withdrawn prior to this test to avoid ongoing HPA suppression or falsely elevated cortisol levels in the case of GCs that are detected by the cortisol assay. In patients believed to be at high risk of adrenal crisis without GC treatment, dexamethasone can be used. Dexamethasone would be associated with suppression of the baseline cortisol level, but ACTH-stimulated cortisol levels should reflect endogenous production since dexamethasone typically does not cross-react with cortisol assays.

^‡^Exogenous estrogen therapy increases serum cortisol; therefore, cortisol levels will not be reliable in the setting of estrogen use.

If AS is suspected, biochemical testing of the HPA axis should be considered after GC treatment has been reduced to a physiologic dose. Given the ease and practicality of a first morning cortisol measurement, it should be considered for the initial screening of patients at risk for AS. The test should be performed at 8:00 am or earlier given that cortisol levels decline throughout the day with natural circadian rhythm, and both evening and morning GC doses should be held prior to testing (see Table 8) [91]. If the 8:00 am cortisol value is below the normal laboratory reference range, AS is likely present and further GC withdrawal should occur only once testing has normalized. It is important to note that the specificity of the first morning cortisol test approaches 100% if a very low cut-off value (<85-112 nmol/L) is used. However, the sensitivity of this test is poor (~60%) [121]. Therefore, a normal cortisol value does not rule out the presence of AS. If a patient has signs or symptoms of AS and requires further testing, then referral to an endocrinologist should be considered. Clinicians must be aware that exogenous estrogen therapy, which affects cortisol-binding globulin levels, increases serum cortisol; therefore, the same thresholds for diagnosing AS do not apply in the setting of estrogen use.

The insulin tolerance test (ITT) is the definitive test for evaluation of the HPA axis, but performing this test is complicated and risky for patients since insulin is administered to achieve hypoglycemia. The ITT is contraindicated in children secondary to the risks of hypoglycemia on the pediatric brain. Therefore, in the setting of a normal morning cortisol result and the presence of AS symptoms, the low-dose adrenocorticotropic hormone (ACTH) stimulation test should be performed to confirm the diagnosis since it is a sensitive and specific test for AS [122-124]. The low-dose ACTH stimulation test involves IV administration of 1 μg of cosyntropin with measurements of baseline and stimulated serum cortisol levels to assess the function of the HPA axis. Cortisol levels are expected to peak between 20–30 min after cosyntropin injection, hence, cortisol measurements are recommended at 15–20 min and 30 min [124]. Many protocols also recommend measuring cortisol at 60 min. A peak cortisol of <500 nmol/L is diagnostic of AS, with both a sensitivity and specificity of approximately 90% [122-124] (note that a lower peak cortisol cut-off value may be required in neonates).

#### Growth in children

For children receiving GC therapy, growth should be monitored every 6 months (ideally by using stadiometry measurements) and measurements should be plotted on an appropriate growth curve (Table 5). If, after 6 months, growth velocity appears to be inadequate, the physician should consider all possible etiologies, including AS, as well as referral to an endocrinologist [91]. It is also important to rule out malnutrition as a cause of poor growth [9,119].

#### CV risk and dyslipidemia

There are currently no evidence-based guidelines for the monitoring of dyslipidemia and CV risk in patients using corticosteroid therapy. The authors recommend assessment of lipid profile at baseline, 1-month after initiating systemic GC therapy and then every 6–12 months thereafter (Table 5). Ten-year CV risk should also be assessed using the Framingham Risk Score (FRS) (https://www.cvdriskchecksecure.com/FraminghamRiskScore.aspx), and lipid targets and treatment should be based on the FRS (see Table 9 for Canadian Cardiovascular Society recommendations) [125].

**Table 9:** **Canadian Cardiovascular Society guidelines for CVD prevention and dyslipidemia management: treatment thresholds and targets based on Framingham Risk Score (FRS) **[125]

**Risk level**	**Initiate therapy if:**	**Primary target LDL-C**	**Alternate target**
**High**	• Consider treatment in all	• ≤2 mmol/L, or	• Apo B ≤0.8 g/L
**FRS ≥20%**		• ≥50% ↓ in LDL-C	• Non HDL-C ≤2.6 mmol/L
**Intermediate**	• LDL-C ≥3.5 mmol/L	• ≤2 mmol/L,or	• Apo B ≤0.8 g/L
**FRS = 10%-19%**	• For LDL-C <3.5 consider if:	• ≥50% ↓ in LDL-C	• Non HDL-C ≤2.6 mmol/L
	→ Apo B ≥1.2 g/L, or		
	→ Non-HDL-C ≥4.3 mmol/L		
**Low**	• LDL-C ≥5.0 mmol/L	• ≥50% ↓ in LDL-C	
**FRS <10%**	• Familial hypercholesterolemia		

Adapted from Anderson et al., 2013 [125].

*FRS* Framingham Risk Score, *HDL-C* high-density lipoprotein cholesterol, *LDL-C*, low-density lipoprotein cholesterol, *apo B* apolipoprotein B.

#### Hyperglycemia/diabetes

All patients should be educated about the classic signs and symptoms of hyperglycemia (polyuria, polydipsia, unexplained weight loss) so that they are screened for steroid-induced diabetes if symptoms arise. In adults, monitoring of glycated hemoglobin (A1C), fasting plasma glucose (FPG), 2-hour plasma glucose (2-h PG) (using a 75-g oral glucose tolerance test [OGTT]), or casual PG (any time of the day without regard to the interval since the last meal) are recommended (Table 5), although FPG, casual PG, and A1C may be less sensitive for diagnosing diabetes. If blood glucose or A1C is abnormal at baseline, then home blood glucose monitoring is also recommended.

Glucose investigations should be repeated after starting GC therapy. In patients taking prednisolone, some experts have recommended that blood glucose be monitored within 8 hours of the first dose (i.e., in the afternoon if once-daily prednisolone is administered in the morning) [37]. The 2013 Canadian Diabetes Association (CDA) guidelines recommend that glycemic parameters be monitored for at least 48 hours after initiation of GC therapy, regardless of whether or not the patient has diabetes [38]. Guidelines for blood glucose monitoring post-transplant suggest weekly monitoring for four weeks after transplant, followed by blood glucose checks at 3 and 6 months post-transplant, then annually thereafter [126]. A diagnosis of diabetes is confirmed if A1C is ≥6.5% (in adults), FPG is ≥7.0 mmol/L, 2-hour PG is ≥11.1 mmol/L or if casual PG is ≥11.1 mmol/L and the patient has classic symptoms of diabetes [38].

In the absence of screening guidelines for GC-induced diabetes in children, the authors recommend that physicians be aware of the risk of hyperglycemia in children receiving long-term supraphysiological GC doses and, at a minimum, screen for classic symptoms [98]. An annual FPG should also be considered.

In children presenting with symptoms suggestive of diabetes, FPG should be performed. If FPG is not diagnostic of diabetes in those with symptoms, OGTT is recommended. The diagnostic criteria for diabetes in children are the same as for adults [38]. More frequent screening of glucose parameters should be considered in children who are at potentially higher risk of developing hyperglycemia or diabetes, such as transplant recipients, obese patients, or those with conditions such as ALL or nephrotic syndrome. Annual OGTT is recommended in children who are very obese and/or who have multiple risk factors for type 2 diabetes since this test may be associated with higher detection rates.

#### Ophthalmologic examination

Patients on low-to-moderate doses of systemic corticosteroids for more than 6–12 months should undergo annual examination by an ophthalmologist (Table 5). An earlier examination is required in patients with symptoms of cataracts (namely blurred vision), however, this is generally not considered an ocular emergency that requires urgent treatment.

Early referral for monitoring of intra-ocular pressure (glaucoma) is recommended in patients at higher risk of developing steroid-induced glaucoma, such as those with a personal or family history of open angle glaucoma, diabetes mellitus, high myopia, or connective tissue disease (especially rheumatoid arthritis).

### Strategies for the prevention and management of GC-induced adverse events

#### General strategies

To minimize the occurrence of steroid-induced AEs, the lowest effective GC dose should be prescribed for the minimum period of time required to achieve treatment goals (Table 10). If possible, consideration should be given to once-daily, morning administration and/or intermittent or alternate-day dosing. Any pre-existing comorbid conditions that may increase the risk of GC-induced AEs should be treated prior to corticosteroid initiation, and patients should be instructed to avoid contact with persons that have infections, such as shingles, chickenpox or measles. Patients should also be advised to carry a steroid treatment card and wear a medical identification tag, and to adopt lifestyle habits that may help minimize the risk of excessive weight gain with GC use, such as participation in regular physical activity and following a healthy, low-calorie diet.

**Table 10:** General strategies for the prevention of GC-induced AEs


• Treat pre-existing comorbid conditions that may increase risk of GC-associated AEs
• Prescribe lowest effective GC dose for minimum period of time required to achieve treatment goals
• Administer as single daily dose (given in the morning), if possible
• Consider intermittent or alternate-day dosing, if possible
• Use GC-sparing agents whenever possible (e.g., omalizumab in severe asthma, azathioprine/cyclophosphamide in vasculitis, methotrexate in rheumatoid arthritis)
• Advise patients to:
▪ Carry a steroid treatment card
▪ Seek medical attention if they experience mood or behavioural changes
▪ Avoid contact with persons that have infections, such as shingles, chickenpox, or measles (unless they are immune)
▪ Not discontinue GC therapy abruptly unless advised to do so by their physician
▪ Adopt lifestyle recommendations to minimize the risk of weight gain or other AEs:
▫ Eat a healthy balanced diet, including adequate calcium intake
▫ Smoking cessation
▫ Reduction in alcohol consumption
▫ Regular physical activity
• Regularly monitor for signs/symptoms of AEs

*GC* glucocorticoid, *AEs* adverse events.

Finally, whenever possible, GC-sparing agents should be considered. In patients with severe asthma, for example, use of the anti-immunoglobulin E (IgE) monoclonal antibody, omalizumab, has been shown to reduce the occurrence of asthma exacerbations requiring systemic corticosteroid therapy and to improve symptoms and asthma-related quality of life [127]. At present, omalizumab is reserved for patients with difficult to control asthma who have documented allergies and whose asthma symptoms remain uncontrolled despite ICS therapy [128].

### Specific recommendations

#### Osteoporosis (adults)

A number of published guidelines have addressed the prevention and treatment of GC-induced osteoporosis in adults [113-117,129-131]. According to the American College of Rheumatology (ACR), adults at low- to medium-risk of fracture (10-year risk of major osteoporotic fracture <20%) exposed to ≥7.5 mg/day of prednisone or equivalent for ≥3 months should be treated with pharmacologic therapy (see Table 11). All patients at high-risk of fracture (10-year risk of major osteoporotic fracture >20%) should receive pharmacologic treatment, irrespective of whether or not they are on GC therapy [115].

**Table 11:** ACR pharmacological recommendations for the prevention and management of GC-induced osteoporosis in adults* [115]

**Postmenopausal women and men age** ≥**50 years starting GC therapy of** ≥**3 months’ duration, or prevalent GC therapy of ≥3 months’ duration**	
**Low-risk (10-year fracture risk < 10%)**	• GC dose < 7.5 mg/day of prednisone or equivalent:
	→ no pharmacologic treatment
	• GC dose ≥ 7.5 mg/day of prednisone or equivalent:
	→ alendronate, risedronate or zoledronic acid
**Medium-risk (10-year fracture risk = 10-20%)**	• GC dose < 7.5 mg/day of prednisone or equivalent:
	→ alendronate or risedronate
	• GC dose ≥ 7.5 mg/day of prednisone or equivalent:
	→ alendronate, risedronate or zoledronic acid
**High-risk (10-year fracture risk > 20%)**	• Any dose or duration of GCs justifies initiating prescription therapy
	▪ If GC dose < 5 mg/day of prednisone or equivalent for ≤ 1 month:
	→ alendronate, risedronate, or zoledronic acid
	▪ If GC dose ≥ 5 mg/day of prednisone or equivalent for ≤ 1 month or any GC dose for > 1 month:
	→ alendronate, risedronate, zoledronic acid or teriparatide^†^
**Premenopausal women and men < 50 years with a history of fragility fracture**	
**GC duration: 1–3 months**	
*Non-childbearing potential:*	• If prednisone (or equivalent) ≥ 5 mg/day: alendronate or risedronate
	• If prednisone (or equivalent) ≥ 7.5 mg/day: zoledronic acid
*Childbearing potential:*	• No consensus
**GC duration: >3 months**	
*Non-childbearing potential:*	• Any dose: alendronate, risedronate, zoledronic acid, teriparatide
*Childbearing potential:*	• If prednisone (or equivalent) < 7.5 mg/day: no consensus
	• If prednisone (or equivalent) ≥ 7.5 mg/day: alendronate, risedronate, teriparatide^†^

*See text for guidelines related to children.

^†^In clinical practice, teriparatide is generally reserved for bisphosphonate treatment failures (i.e., new vertebral fracture or ≥2 non-vertebral fractures after adherence to 12 months of bisphosphonate treatment).

Most guidelines and evidence support the use of bisphosphonates and teriparatide as first-line therapy for GC-induced osteoporosis in adults. A number of studies have demonstrated that the bisphosphonates alendronate, risedronate and zoledronic acid are effective for the prevention and treatment of GC-induced bone loss [132-138], although their long-term efficacy on fractures is not well established [132]. Teriparatide has been shown to be effective in improving BMD and reducing vertebral fractures in patients with GC-induced osteoporosis [139-141]. The ACR recommendations for the use of teriparatide and bisphosphonates are shown in Table 11[115].

Although other therapies such as calcitonin, raloxifene and denosumab may also play a role in the management of GC-induced osteoporosis in adults, they are not currently recommended as first-line therapy. Calcitonin has been found to prevent lumbar spine bone loss in the setting of GC use, but the same protection has not been observed at the femoral neck or with respect to fracture risk [142]. The European Medicines Agency (EMA) recently completed a review of the benefits and risks of calcitonin-containing medicines and concluded that there is evidence of a small, increased risk of cancer (0.7-2.4%) with long-term use of these agents [143]. Therefore, given this risk as well as its lack of efficacy in reducing fracture risk, calcitonin is not recommended as first-line therapy for GC-induced osteoporosis. However, it may be considered when bisphosphonates are contraindicated or in those patients who are intolerant to oral or IV bisphosphonates. Due to its analgesic effect, calcitonin can also be considered in patients who have sustained an acute fracture.

A study of postmenopausal women on ≤10 mg/day of prednisone (or equivalent) for ≥6 months demonstrated that treatment with raloxifene for 1 year improved spine and total hip (but not femoral neck) BMD [144]. However, the generalizability of these findings are limited since the study cohort was predominantly Asian and did not include patients on high-dose GC therapy. Furthermore, as a selective estrogen receptor modulator, raloxifene use for osteoporosis prevention and treatment is limited to the postmenopausal female population.

In animal models, denosumab has been shown to prevent steroid-induced bone loss and improve bone strength [145]. A phase 2 trial also found that denosumab improved lumbar spine BMD in patients with rheumatoid arthritis treated with corticosteroids and bisphosphonates [146]. The phase 3 FREEDOM trial found denosumab to be associated with a slightly increased risk of cellulitis [147], although the 2-year extension trial found no increased risk with longer term treatment [148].

In addition to pharmacologic therapy, current guidelines for GC-induced osteoporosis in adults recommend preventive measures such as smoking cessation, reduced alcohol consumption, participation in weight-bearing and strength-building exercises, falls risk assessment, and calcium and vitamin D supplementation [113-117,129-131]. Cochrane investigators reviewed the available data on calcium and vitamin D use in GC-treated patients and found that supplementation prevented bone loss at the lumbar spine and forearm, but had no effect on femoral neck BMD or fracture incidence [149]. Adults on high-dose GC therapy should be taking 1200 mg/day of elemental calcium in divided doses and 800–2000 IU of vitamin D daily [113,117].

#### Osteoporosis (children)

There are currently no evidence-based guidelines for the prevention and treatment of GC-induced osteoporosis in children. General measures are similar to those described above and include: using the lowest effective GC dose possible for the shortest period of time; proper nutrition and maintenance of a healthy weight; promotion of weight-bearing exercise; vitamin D supplementation to achieve at least 50 nmol/L, and ideally 75 nmol/L [150,151]; calcium supplementation (if diet is inadequate to achieve the current, recommended dietary allowance) [150]; and the use of GC-sparing agents when possible. Most of the studies examining bisphosphonate use in GC-treated children have been observational in nature and have utilized the intravenous (IV) preparation, pamidronate [152]. Some evidence suggests that bisphosphonate therapy increases BMD, promotes reshaping and relieves back pain from previously fractured vertebral bodies, and is safe and well-tolerated in children with secondary osteoporosis [152-155], although long-term safety and efficacy data is still required. Currently, experts recommend consideration of bisphosphonate therapy in children with evident bone fragility associated with reductions in BMD parameters, particularly if there is a persistence of risk factors and, thereby, less likelihood of spontaneous BMD restitution and growth-mediated reshaping of vertebral bodies [153].

#### Osteonecrosis

Initial treatment for osteonecrosis includes bed rest and non-steroidal or other analgesics to relieve pain. For patients with early stage or less advanced osteonecrosis, joint-preserving strategies, such as reducing weight-bearing activities and core decompression (with or without marrow transplantation), have been utilized with varying levels of success. For more advanced disease, femoral head or total hip replacement surgery is usually required [16]. Since these replacements generally have a 10-year lifespan, strategies that delay the need for surgery are desired. Some evidence suggests that treatment with alendronate may reduce the risk of bone collapse and delay the need for surgery [156,157]. However, a recent randomized, controlled trial found no benefit of alendronate vs. placebo in patients with osteonecrosis [158]. In children with osteonecrosis in the leukemia setting, IV pamidronate has been associated with significant improvements in pain and mobility [159,160].

#### Hyperglycemia and diabetes

Glycemic targets for patients with GC-induced diabetes should be individualized, but for most patients, FPG and 2-h PG targets of 4.0-7.0 mmol/L and 5–10 mmol/L, respectively, are recommended (see Table 12) [38]. When possible, referral to a multidisciplinary diabetes team should be considered. Initial management involves appropriate lifestyle modification strategies; if targets are not met with these modifications, pharmacotherapy is recommended, and the same spectrum of glucose-lowering medications is used for GC-induced diabetes as is used for pre-existing type 2 diabetes. If blood glucose levels are <15 mmol/L, then glucose control can likely be achieved with non-insulin therapies such as metformin, dipeptidyl peptidase-4 (DPP-4) inhibitors, sulfonylureas, meglitinides, or glucagon-like peptide-1 (GLP-1) agonists (Table 12). If a sulfonylurea is selected, it is important to consider both the dosing frequency of the GC as well as the duration of action of the insulin secretagogue. Sulfonylureas with shorter half-lives (i.e., glyburide, gliclazide or repaglinide) are more suitable for patients using once-daily prednisone as they can be dosed once-per-day along with the GC. Agents with longer half-lives (e.g., gliclazide MR or glimepiride) may be more suitable for those using dexamethasone or shorter-acting GCs that are administered more than once daily.

**Table 12:** Glycemic targets and treatment recommendations for GC-induced diabetes in adults

**Glycemic targets for most patients **[38]**:**			
**A1C: ≤7.0 %**		**FPG: 4.0–7.0 mmol/L**	**2-hr PPG: 5.0–10.0 mmol/L**
**Management:**			
*Lifestyle interventions:*			
• Initiate nutrition therapy and physical activity; if BG targets not met, initiate pharmacotherapy			
*Pharmacotherapy:*			
If BG < 15 mmol/L:	Non-insulin therapies →	• Metformin	
		• Insulin secretagogues	→ If using once-daily prednisone, use shorter-acting agents (e.g., glyburide, gliclazide, repaglinide) dosed once-daily with prednisone
			→ If using dexamethasone or shorter-acting agents > once/day, use longer-acting agents (e.g., gliclazide MR, glimepiride)
		• DPP-4 inhibitor	
		• GLP-1 agonist	
If BG < 15 mmol/L vs. >15 mmol/L	Insulin	→ Starting dose: 0.15-0.3 units/kg/day	
		→ If using once-daily prednisone in the morning, FPG less affected but BG will be higher later in the day:	
		• Initiate intermediate-acting insulin (N or NPH) or a premixed combination of intermediate- and fast-acting insulin, administered in the morning	
		• Add evening insulin if FPG is elevated	
		→ If using dexamethasone or shorter-acting agents > once/day, BG likely to be affected throughout the entire day:	
		• Use intermediate-acting insulin twice daily or long-acting insulin (detemir, glargine)	
		• Fast-acting insulin at mealtimes can be used in combination with intermediate- and long-acting insulin	
	Metformin	→ Often recommended in combination with insulin	
**Caution: If reducing the GC dose, adjust diabetes medications to avoid hypoglycemia**			

*A1C* glycated hemoglobin, *FPG* fasting plasma glucose, *PPG* postprandial plasma glucose, *BG* blood glucose; *DPP-4* dipeptidyl peptidase-4, *GLP-1* glucagon-like peptide-1.

If blood glucose levels are >15 mmol/L, then insulin is usually required to achieve glycemic control. In the absence of a contraindication, metformin is often recommended in combination with insulin (Table 12). A reasonable starting dose for insulin is 0.15-0.3 units/kg/day. With once-daily morning administration of prednisone, fasting glucose may be unaffected, but blood glucose will be higher later in the day. If this occurs, then an intermediate-acting insulin (such as N or NPH) or a premixed combination of intermediate- and fast-acting insulin can be initiated in the morning. If blood glucose is elevated in the morning as well, then an evening insulin dose may also be required. If shorter-acting GCs are administered more than once per day, or if dexamethasone is used, then both fasting and non-fasting glucose levels are likely to be affected. In this case, twice-daily intermediate-acting insulin or long-acting insulin, such as detemir or glargine, are recommended; fast-acting insulin may also be required at mealtimes. In order to prevent hypoglycemia, it is important to remember to adjust diabetes medications if GC doses are reduced.

The treatment of GC-induced diabetes in children is best accomplished through the combined efforts of a multidisciplinary pediatric diabetes healthcare team [98]. As with adults, lifestyle interventions should be initiated; if glycemic targets are not met with these modifications, insulin must be considered. Many of the other glucose-lowering agents used in adult patients with type 2 diabetes have not been licensed for use in the pediatric population and may be contraindicated in children with complex medical issues [98]. Until the safety and efficacy of these medications in children are established, they cannot be recommended for routine clinical use in this population.

#### Adrenal suppression (AS)

To minimize the risk of developing AS, it is important to consider the relative suppressive effects of the various GCs (based on potency and duration of action) prior to initiating therapy (see Table 3). The lowest effective dose should be utilized for treatment of the underlying condition and the dose should be re-evaluated regularly to determine if further reductions can be instituted. If possible, the GC should be administered once-daily in the morning.

Currently, evidence-based recommendations are lacking for withdrawal of high-dose GC treatment and management of individuals with biochemical evidence of AS. If high-dose GC therapy is no longer required, then GC doses can be reduced relatively quickly from pharmacologic to physiologic doses. Examples of withdrawal regimens for both adults and children are provided in Tables 13 and 14, respectively. These tables present modest, but safe, approaches to GC withdrawal and assume that the clinician has access to testing. However, in the absence of evidence-based guidelines, some physicians may choose to withdraw GC therapy gradually without testing. Regardless of the withdrawal regimen chosen, clinicians need to be aware of the symptoms of AS and to slow the withdrawal regimen should these symptoms arise.

**Table 13:** Prednisone tapering regimen for adults

	
1. Reduce dose by 2.5- to 5.0-mg decrements every 3–7 days until physiologic dose (5 to 7.5 mg of prednisone per day) is reached; slower tapering of GC therapy may be recommended if risk of disease relapse is a concern	
2. Switch to hydrocortisone 20 mg once-daily, given in the morning	
3. Gradually reduce hydrocortisone dose by 2.5 mg over weeks to months	
4. Discontinue/continue hydrocortisone based on assessment of morning cortisol:	
< 85 nmol/L:	HPA-axis has not recovered
	→ continue hydrocortisone
	→ re-evaluate patient in 4–6 weeks
85-275 nmol/L:	Suspicious for AS
	→ Continue hydrocortisone
	→ Further testing of HPA axis or re-evaluate in 4–6 weeks
	→ If further evaluation of HPA axis is selected:
	▪ ITT (gold-standard but not widely available)
	▪ ACTH stimulation testing (see below)
276-500 nmol/L:	HPA-axis function is likely adequate for daily activities in a non-stressed state, but may be inadequate for preventing adrenal crisis at times of stress or illness
	→ Discontinue hydrocortisone
	→ Monitor for signs & symptoms of AS
	→ Consider further evaluation of HPA axis to determine if function is also adequate for stressed states or consider empiric therapy with high-dose steroids during times of stress
> 500 nmol/L:	HPA axis is intact
	→ discontinue hydrocortisone
**↓**	
***If ACTH stimulation testing is performed and:***	
Peak cortisol rises to > 500 nmol/L:	HPA axis intact and GC can be discontinued
Peak cortisol < 500 nmol/L:	Steroids required at times of stress and illness until normal ACTH response is noted

*AS* adrenal suppression, *GC* glucocorticoid, *HPA* hypothalamic-pituitary-adrenal, *ACTH* adrenocorticotropic hormone, *ITT* insulin tolerance test.

**Note:** Exogenous estrogen therapy increases serum cortisol; therefore, the same thresholds for diagnosing AS do not apply in the setting of estrogen use.

**Table 14:** Prednisone tapering regimen for children

	
1. Taper GC dose as guided by underlying condition until 30 mg/m^2^/day of hydrocortisone equivalent is reached (if taper not required for underlying condition, reduce to 30 mg/m^2^/day)	
2. Then taper by 10-20% every 3–7 days until patient is on physiological GC dose (8–10 mg/m^2^/day hydrocortisone equivalent)	
3. Switch to hydrocortisone 8–10 mg/m^2^/day, given in the morning	
4. Discontinue/continue hydrocortisone based on assessment of morning cortisol:	
< 171 nmol/L*:	HPA axis has not recovered
	→ continue daily hydrocortisone
	→ continue stress hydrocortisone as needed
	→ re-evaluate patient in 4–6 weeks
> 500 nmol/L:	HPA axis is intact
	→ discontinue daily and stress hydrocortisone
171*-500 nmol/L:	Sufficient GC production for day-to-day functioning†
	Further evaluation required to determine if stress dosing required:
	→ discontinue daily hydrocortisone
	→ continue stress dosing as needed
	→ low-dose ACTH stimulation testing
**↓**	
***If with ACTH testing:***	
Peak cortisol > 500 nmol/L:	HPA axis is intact and GC can be discontinued
Peak cortisol < 500 nmol/L:	Steroids required at times of stress and illness until normal ACTH response is noted

GC: glucocorticoid; HPA: hypothalamic-pituitary-adrenal; ACTH: adrenocorticotropic hormone.

*If lab norm for morning cortisol is >171 nmol/L, use lab norm.

^†^If symptomatic despite normal first morning cortisol, continue daily and stress hydrocortisone and contact pediatric endocrinologist.

Screening tests should be considered to assess adrenal function as GC therapy is being withdrawn. Screening should occur before tapering to less than a physiologic dose (Tables 13 and 14) [161,162]. When possible, screening should occur at least 1 week after the dose has been tapered to a once-daily physiological dose (preferably hydrocortisone, which has a shorter half-life).

Symptomatic AS should be treated with daily physiologic replacement doses of GC plus “stress doses” during physiological stress (intercurrent illness, injury or surgery) (see Tables 15 and 16). This treatment model replicates the physiological response of the healthy adrenal gland in order to prevent an adrenal crisis. Theoretically, an individual with biochemical evidence of AS in the absence of symptoms is also at risk of adrenal crisis and should receive “stress doses” of GC during physiological stress, with or without daily physiologic GC. To our knowledge, there is no evidence to support or refute this practice. The safest approach would be to treat asymptomatic patients with biochemical evidence of AS no differently than those with symptomatic AS. At a minimum, these patients should be aware of their diagnosis and be provided with an information card that outlines the need to receive GC “stress doses” during critical illness or surgery (see Tables 15 and 16).

**Table 15:** **Recommendations for the management of AS in children **[91]

	
**1. Stress steroids during periods of physiological stress**	
Adrenal crisis/critical illness*:	Hydrocortisone injection (Solu-Cortef) 100 mg/m^2^ (max. 100 mg) IV/IM stat with saline volume expansion, followed by 25 mg/m^2^ q 6 hours (max. 25 mg q 6 hours); call endocrinologist on call
Surgery*:	Hydrocortisone injection (Solu-Cortef) 50–100 mg/m^2^ IV (max 100 mg) pre-operatively, then 25 mg/m^2^ q 6 hours (max 25 mg q 6 hours); call endocrinologist on call
*Illness or fever:*	20 mg/m^2^/day hydrocortisone equivalent, divided BID or TID
*Fever >38.5°C or vomiting:*	30 mg/m^2^/day hydrocortisone equivalent, divided TID
*Unable to tolerate orally:*	Hydrocortisone must be administered parenterally as Solu-Cortef, 25 mg/m^2^/dose q 6 hours IV or q 8 hours IM
**2. ± Daily physiologic dose of hydrocortisone (8–10 mg/m**^**2**^**/day)**	
**3. Patient/family education**	
– Stress steroid dosing	
– Emergency medical contact information in case of illness	
4. Information card*	
**5. Consider medical identification tag**	

IV: intravenous; IM: intramuscular; BID: twice daily; TID: three times daily; QID: four times daily; q: every.

*At a minimum, symptomatic patients require an information card and stress dosing during critical illness and surgery.

Reproduced from Ahmet et al., 2011 [91].

**Table 16:** Recommendations for the management of AS in adults

**Medical or surgical stress**		**Examples of stress**	**Recommended GC dose in addition to usual GC dose**
*Minor*	Procedure/surgery:	• Inguinal hernia repair	Hydrocortisone 25 mg or equivalent pre-op
	Medical illness:	• Mild febrile illness	Hydrocortisone 25 mg/day or equivalent*
		• Mild-moderate nausea/vomiting	
		• Gastroenteritis	
*Moderate*	Surgery:	• Open cholecystectomy	Hydrocortisone 50–75 mg/day or equivalent from pre-op until 1–2 days after procedure*
		• Segmental colon resection	
		• Total joint replacement	
		• Abdominal hysterectomy	
	Medical illness:	• Significant febrile illness	Hydrocortisone 50–75 mg/day or equivalent during illness*
		• Severe gastroenteritis	
*Severe*	Surgery:	• Pancreatoduodenectomy	Hydrocortisone 100–150 mg/day or equivalent from pre-op until 2–3 days after procedure*
		• Esophagogastrectomy	
		• Liver resection	
		• Surgery involving cardiopulmonary bypass	
	Medical illness:	• Pancreatitis	Hydrocortisone 100–150 mg/day or equivalent during illness*
*Critical illness*			Hydrocortisone 50–100 mg IV q 6–8 hours then taper as clinical status improves

*If a corticosteroid with a short half-life (e.g., hydrocortisone) is given, then GC dose should be divided into 2–3 doses/day.

Adapted from: Coursin, Wood, 2002 [161] and Salem et al., 1994 [162].

Consideration should be made to educate patients about the risk of AS if they have been treated with GC therapy within the last year, but have not had testing to rule out AS. In the event of severe illness or surgery, stress dose steroids should be considered to prevent adrenal crisis.

#### Growth

The potency of dexamethasone and betamethasone in suppressing growth has been shown to be nearly 18 times higher than that of prednisolone [163]. Therefore, to reduce the risk of growth suppression in children, lower potency agents, such as prednisolone, should be used whenever possible. Consideration should also be given to alternate-day dosing (if possible) since evidence suggests that the use of lower doses of prednisolone (10–15 mg/day or < 0.5 mg/kg/day single dose) on alternate days does not significantly slow growth velocity [9].

There is currently insufficient evidence to support the use of recombinant human growth hormone (rhGH) for the treatment/prevention of GC-induced growth suppression. There has been some evidence of short-term benefits on growth velocity with rhGH therapy [164], however further study, including evaluation of final adult height, is required.

#### Gastrointestinal (GI) events

Consideration can be given to the use of proton pump inhibitors (PPIs) for GI protection in GC users at high-risk of GI bleeding or peptic ulcers, such as those using NSAIDS, patients with a history of ulcers or GI bleeding, and those with serious comorbidities (i.e., advanced cancer) [1].

#### Cutaneous events: Red striae

Although treatment of red striae is often disappointing, some success has been noted with topical vitamin A 0.1% cream, flashlamp-pumped pulsed dye lasers, and a combination of pulsed dye laser and Thermage (a non-ablative radiofrequency device) [44]. To help reduce the risk of striae, patients initiating systemic corticosteroid therapy should be advised to follow a low-calorie diet.

## Conclusions

Systemic corticosteroids are widely used to treat a variety of autoimmune and inflammatory disorders. Despite the benefits of these agents, their prolonged use (particularly at high doses) is associated with potentially serious AEs affecting the musculoskeletal, endocrine, CV, and central nervous systems as well as the GI tract. Many of these side effects can be minimized through careful patient monitoring and implementation of preventive measures, including the use of lower potency agents and the lowest effective dose required for management of the underlying condition.

Patients should be informed about the AEs associated with systemic corticosteroid use and should be advised on lifestyle modification strategies that may help reduce the risk of these events. Patients should also be instructed to seek medical attention if they experience signs and symptoms of steroid-related AEs and should be advised to carry a steroid treatment card that can be shown to all healthcare professionals involved in their care and management. Differences in the monitoring and care of adults versus children should also be noted, particularly in terms of GC-associated complications related to growth, AS and osteoporosis.

## Abbreviations

A1C: Glycated hemoglobin; ACTH: Adrenocorticotropic hormone; AE: Adverse event; AF: Atrial fibrillation; ALL: Acute lymphoblastic leukaemia; apo B: Apolipoprotein B; AS: Adrenal suppression; BG: Blood glucose; GI: Gastrointestinal; BMC: Bone mineral content; BMD: Bone mineral density; BMI: Body mass index; CBC: Complete blood count; CDA: Canadian Diabetes Association; CI: Confidence interval; COPD: Chronic obstructive pulmonary disease; CSCR: Central serous chorioretinopathy; CVD: Cardiovascular disease; DPP-4: Dipeptidyl peptidase-4; DMARD: Disease-modifying antirheumatic drug; FPG: Fasting plasma glucose; FRAX: Fracture Risk Assessment Tool; FRS: Framingham Risk Score GC, glucocorticoid; GI: Gastrointestinal; GLP-1: Glucagon-like peptide-1; HCTZ: Hydrochlorothiazide; HDL-C: High-density lipoprotein cholesterol; HPA: Hypothalamic-pituitary-adrenal; ICU: Intensive care unit; INR: International normalized ratio; ITT: Insulin tolerance test; LDL-C: Low-density lipoprotein cholesterol; NSAIDS: Non-steroidal anti-inflammatory drugs; OGTT: Oral glucose tolerance test; OR: Odds ratio; PG: Plasma glucose; PPI: Proton pump inhibitor; RR: Relative risk; SLE: Systemic lupus erythematosus; TC: Total cholesterol; TG: Triglycerides; TIA: Transient ischemic attack.

## Competing interests

Dr. Alexandra Ahmet has received honoraria for continuing education from Nycomed. Dr. Harold Kim has received consulting fees and honoraria for continuing education from AstraZeneca, Pfizer, Merck Frosst, Novartis, and Takeda. Dr. Leanne Ward has received consultant fees from Novartis Pharmaceuticals and Amgen in the past 5 years. She has no non-financial competing interests to declare. Dr. Albert Cohen has received consulting fees and honoraria from Janssen and AbbVie. Dr. Richard Leigh has received consulting fees and honoraria for continuing education from AstraZeneca, GlaxoSmithKline, Novartis and Takeda. Dr. Jacques P. Brown has received research grants from Abbott, Amgen, Bristol-Myers Squibb, Eli Lilly, Merck, Novartis, Pfizer, Roche, Sanofi-aventis, Servier, Takeda, and Warner Chilcott. He has received consulting fees or other remuneration from Amgen, Eli Lilly, Merck, Novartis, Sanofi-aventis, and Warner Chilcott, and has served on the speaker’s bureau for Amgen, Eli Lilly, and Novartis. Dr. Preetha Krishnamoorthy has received honoraria for continuing medical education from Takeda (previously Nycomed).

Dr. Dora Liu and Dr. Efrem Mandelcorn have no competing interests to declare.

## Authors’ contributions

DL, AA, HK, LW, RL, and EM contributed to the conception, drafting and writing of the manuscript and to revising it for important intellectual content. AC, PK and JB contributed to the revision and intellectual content of this manuscript. All authors read and approved the final manuscript.

## References

[B1] National Institute for Health and Clinical Excellence (NICE) Clinical Knowledge Summaries: Corticosteroids - Oral 2012 NICE [http://www.cks.nhs.uk/corticosteroids_oral], Accessed February 20, 2013

[B2] Singh N Rieder MJ Tucker MJ Mechanisms of glucocorticoid-mediated antiinflammatory and immunosuppressive action Paed Perinatal Drug Ther 2004 6 107 115

[B3] Newton R Leigh R Giembycz MA Pharmacological strategies for improving the efficacy and therapeutic ratio of glucocorticoids in inflammatory lung diseases Pharmacol Ther 2010 125 286 327 10.1016/j.pharmthera.2009.11.003 19932713

[B4] Coutinho AE Chapman KE The anti-inflammatory and immunosuppressive effects of glucocorticoids, recent developments and mechanistic insights Mol Cell Endocrinol 2011 335 2 13 10.1016/j.mce.2010.04.005 20398732 PMC3047790

[B5] Croxtall JD van Hal PT Choudhury Q Gilroy DW Flower RJ Different glucocorticoids vary in their genomic and non-genomic mechanism of action in A549 cells Br J Pharmacol 2002 135 511 519 10.1038/sj.bjp.0704474 11815387 PMC1573139

[B6] Smoak KA Cidlowski JA Mechanisms of glucocorticoid receptor signaling during inflammation Mech Ageing Dev 2004 125 697 706 10.1016/j.mad.2004.06.010 15541765

[B7] Stellato C Post-transcriptional and nongenomic effects of glucocorticoids Proc Am Thorac Soc 2004 1 255 263 10.1513/pats.200402-015MS 16113443

[B8] Furst DE Saag KG Determinants of glucocorticoid dosing Up To Date 2012 2013 http://www.uptodate.com/contents/determinants-of-glucocorticoid-dosing?source=search_result&search=glucocorticoid&selectedTitle=4~150

[B9] Deshmukh CT Minimizing side effects of systemic corticosteroids in children Indian J Dermatol Venereol Leprol 2007 73 218 221 10.4103/0378-6323.33633 17675727

[B10] Da Silva JA Jacobs JW Kirwan JR Boers M Saag KG Inês LB de Koning EJ Buttgereit F Cutolo M Capell H Rau R Bijlsma JW Safety of low dose glucocorticoid treatment in rheumatoid arthritis: published evidence and prospective trial data Ann Rheum Dis 2006 65 285 293 10.1136/ard.2005.038638 16107513 PMC1798053

[B11] Weinstein RS Jilka RL Parfitt AM Manolagas SC Inhibition of osteoblastogenesis and promotion of apoptosis of osteoblasts and osteocytes by glucocorticoids. Potential mechanisms of their deleterious effects on bone J Clin Invest 1998 102 274 282 10.1172/JCI2799 9664068 PMC508885

[B12] Yao W Cheng Z Busse C Pham A Nakamura MC Lane NE Glucocorticoid excess in mice results in early activation of osteoclastogenesis and adipogenesis and prolonged suppression of osteogenesis: a longitudinal study of gene expression in bone tissue from glucocorticoid-treated mice Arthritis Rheum 2008 58 1674 1686 10.1002/art.23454 18512788 PMC3892702

[B13] Manolagas SC Corticosteroids and fractures: a close encounter of the third cell kind J Bone Miner Res 2000 15 1001 1005 10.1359/jbmr.2000.15.6.1001 10841168

[B14] van Staa TP Leufkens HG Cooper C The epidemiology of corticosteroid-induced osteoporosis: a meta-analysis Osteoporos Int 2002 13 777 787 10.1007/s001980200108 12378366

[B15] Kanis JA Johansson H Oden A Johnell O de Laet C Melton LJ III Tenenhouse A Reeve J Silman AJ Pols HA Eisman JA McCloskey EV Mellstrom D A meta-analysis of prior corticosteroid use and fracture risk J Bone Miner Res 2004 19 893 899 10.1359/JBMR.040134 15125788

[B16] Weinstein RS Glucocorticoid-induced osteonecrosis Endocrine 2012 41 183 190 10.1007/s12020-011-9580-0 22169965 PMC3712793

[B17] Kaste SC Karimova EJ Neel MD Osteonecrosis in children after therapy for malignancy Am J Roentgeno 2011 196 1011 1018 10.2214/AJR.10.6073 PMC4700933 21512065

[B18] Barr RD Sala A Osteonecrosis in children and adolescents with cancer Pediatr Blood Cancer 2008 50 2 Suppl 483 485 18064641 10.1002/pbc.21405

[B19] Seamon J Keller T Saleh J Cui Q The pathogenesis of nontraumatic osteonecrosis Arthritis 2012 2012 601763 23243507 10.1155/2012/601763 PMC3518945

[B20] Zhao FC Li ZR Guo KJ Clinical analysis of osteonecrosis of the femoral head induced by steroids Orthop Surg 2012 4 28 34 10.1111/j.1757-7861.2011.00163.x 22290816 PMC6583143

[B21] Fauci AS Braunwald E Kasper DL Hauser SL Longo DL Jameson JL Loscalzo J Harrison’s Principles of Internal Medicine 2008 17 The McGraw-Hill Companies, Inc http://www.amazon.ca/books/dp/0071466339

[B22] Livanou T Ferriman D James VH Recovery of hypothalamo-pituitary-adrenal function after corticosteroid therapy Lancet 1967 2 856 859 12387243 10.1016/s0140-6736(67)92592-5

[B23] Henzen C Suter A Lerch E Urbinelli R Schorno XH Briner VA Suppression and recovery of adrenal response after short-term, high-dose glucocorticoid treatment Lancet 2000 355 542 545 10.1016/S0140-6736(99)06290-X 10683005

[B24] Molimard M Girodet PO Pollet C Fourrier-Réglat A Daveluy A Haramburu F Fayon M Tabarin A Inhaled corticosteroids and adrenal insufficiency: prevalence and clinical presentation Drug Saf 2008 31 769 774 10.2165/00002018-200831090-00005 18707191

[B25] Habib GS Systemic effects of intra-articular corticosteroids Clin Rheumatol 2009 28 749 756 10.1007/s10067-009-1135-x 19252817

[B26] Hengge UR Ruzicka T Schwartz RA Cork MJ Adverse effects of topical glucocorticosteroids J Am Acad Dermatol 2006 54 1 15 10.1016/j.jaad.2005.01.010 16384751

[B27] Ortega E Rodriguez C Strand LJ Segre E Effects of cloprednol and other corticosteroids on hypothalamic-pituitary-adrenal axis function J Int Med Res 1976 4 326 337 1028634 10.1177/030006057600400506

[B28] Nichols T Nugent CA Tyler FH Diurnal variation in suppression of adrenal function by glucocorticoids J Clin Endocrinol Metab 1965 25 343 349 10.1210/jcem-25-3-343 14264259

[B29] Shulman DI Palmert MR Kemp SF Lawson Wilkins Drug and Therapeutics Committee Adrenal insufficiency: still a cause of morbidity and death in childhood Pediatrics 2007 119 e484 e494 10.1542/peds.2006-1612 17242136

[B30] LaRochelle GE Jr LaRochelle AG Ratner RE Borenstein DG Recovery of the hypothalamic-pituitary-adrenal (HPA) axis in patients with rheumatic diseases receiving low-dose prednisone Am J Med 1993 95 258 264 10.1016/0002-9343(93)90277-V 8368224

[B31] Einaudi S Bertorello N Masera N Farinasso L Barisone E Rizzari C Corrias A Villa A Riva F Saracco P Pastore G Adrenal axis function after high-dose steroid therapy for childhood acute lymphoblastic leukemia Pediatr Blood Cancer 2008 50 537 541 10.1002/pbc.21339 17828747

[B32] Curtis JR Westfall AO Allison J Bijlsma JW Freeman A George V Kovac SH Spettell CM Saag KG Population-based assessment of adverse events associated with long-term glucocorticoid use Arthritis Rheum 2006 55 420 426 10.1002/art.21984 16739208

[B33] Fardet L Cabane J Lebbé C Morel P Flahault A Incidence and risk factors for corticosteroid-induced lipodystrophy: a prospective study J Am Acad Dermatol 2007 57 604 609 10.1016/j.jaad.2007.04.018 17582650

[B34] Huscher D Thiele K Gromnica-Ihle E Gromnica-Ihle E Hein G Demary W Dreher R Zink A Buttgereit F Dose-related patterns of glucocorticoid-induced side effects Ann Rheum Dis 2009 68 1119 1124 10.1136/ard.2008.092163 18684744

[B35] Schneiter P Tappy L Kinetics of dexamethasone-induced alterations of glucose metabolism in healthy humans Am J Physiol 1998 275 E806 E813 9815000 10.1152/ajpendo.1998.275.5.E806

[B36] Gurwitz JH Bohn RL Glynn RJ Monane M Mogun H Avorn J Glucocorticoids and the risk for initiation of hypoglycemic therapy Arch Intern Med 1994 154 97 101 10.1001/archinte.1994.00420010131015 8267494

[B37] Burt MG Roberts GW Aguilar-Loza NR Frith P Stranks SN Continuous monitoring of circadian glycemic patterns in patients receiving prednisolone for COPD J Clin Endocrinol Metab 2011 96 1789 1796 10.1210/jc.2010-2729 21411550

[B38] Canadian Diabetes Association Clinical Practice Guidelines Expert Committee Canadian Diabetes Association 2013 clinical practice guidelines for the prevention and management of diabetes in Canada Can J Diabetes 2013 37 Suppl 1 S1 S212 24070926 10.1016/j.jcjd.2013.01.009

[B39] American Diabetes Association Standards of medical care in diabetes — 2012 Diabetes Care 2012 35 Suppl 1 S11 S63 22187469 10.2337/dc12-s011 PMC3632172

[B40] Black RL Oglesby RB von Sallman L Bunim JJ Posterior subcapsular cataracts induced by corticosteroids in patients with rheumatoid arthritis JAMA 1960 174 166 171 10.1001/jama.1960.63030020005014 13801171

[B41] Urban RC Jr Cotlier E Corticosteroid-induced cataracts Surv Ophthalmol 1986 31 102 110 10.1016/0039-6257(86)90077-9 3541262

[B42] Armaly MF Effect of corticosteroids on intraocular pressure and fluid dynamics: The effect of dexamethasone in the normal eye Arch Ophthalmol 1963 70 482 491 10.1001/archopht.1963.00960050484010 14078870

[B43] Haimovici R Gragoudas ES Duker JS Sjaarda RN Eliott D Central serous chorioretinopathy associated with inhaled or intranasal corticosteroids Ophthalmol 1997 104 1653 1660 10.1016/S0161-6420(97)30082-7 9331207

[B44] Schellenberg R Adachi JDR Bowie D Brown J Guenther L Kader T Trope GE Oral corticosteroids in asthma: a review of benefits and risks Can Respir J 2007 14 Suppl C 1C 7C

[B45] Poetker DM Reh DD A comprehensive review of the adverse effects of systemic corticosteroids Otolaryngol Clin North Am 2010 43 753 768 10.1016/j.otc.2010.04.003 20599080

[B46] Conn HO Blitzer BL Nonassociation of adrenocorticosteroid therapy and peptic ulcer N Engl J Med 1976 294 434 479 10.1056/NEJM197602262940905 173997

[B47] Conn HO Poynard T Corticosteroids and peptic ulcer: meta-analysis of adverse events during steroid therapy J Intern Med 1994 236 619 632 10.1111/j.1365-2796.1994.tb00855.x 7989897

[B48] Saag KG Furst DE Major side effects of systemic glucocorticoids Up To Date 2012 2013 http://www.uptodate.com/contents/major-side-effects-of-systemic-glucocorticoids

[B49] Piper JM Ray WA Daugherty JR Griffin MR Corticosteroid use and peptic ulcer disease: role of nonsteroidal anti-inflammatory drugs Ann Intern Med 1991 114 735 740 10.7326/0003-4819-114-9-735 2012355

[B50] Messer J Reitman D Sacks HS Smith H Jr Chalmers TC Association of adrenocorticosteroid therapy and peptic-ulcer disease N Engl J Med 1983 309 21 24 10.1056/NEJM198307073090105 6343871

[B51] Sadr-Azodi O Mattsson F Bexlius TS Lindblad M Lagergren J Ljung R Association of oral glucocorticoid use with an increased risk of acute pancreatitis: a population-based nested case–control study JAMA Intern Med 2013 173 444 449 10.1001/jamainternmed.2013.2737 23440105

[B52] Derk CT DeHoratius RJ Systemic lupus erythematosus and acute pancreatitis: a case series Clin Rheumatol 2004 23 147 151 10.1007/s10067-003-0793-3 15045630

[B53] Wei L MacDonald TM Walker BR Taking glucocorticoids by prescription is associated with subsequent cardiovascular disease Ann Intern Med 2004 141 764 770 10.7326/0003-4819-141-10-200411160-00007 15545676

[B54] Souverein PC Berard A Van Staa TP Cooper C Egberts AC Leufkens HG Walker BR Use of oral glucocorticoids and risk of cardiovascular and cerebrovascular disease in a population based case–control study Heart 2004 90 859 865 10.1136/hrt.2003.020180 15253953 PMC1768386

[B55] van der Hooft CS Heeringa J Brusselle GG Hofman A Witteman JC Kingma JH Sturkenboom MC Stricker BH Corticosteroids and the risk of atrial fibrillation Arch Intern Med 2006 166 1016 1020 10.1001/archinte.166.9.1016 16682576

[B56] Christiansen CF Christensen S Mehnert F Cummings SR Chapurlat RD Sørensen HT Glucocorticoid use and risk of atrial fibrillation or flutter: a population-based, case–control study Arch Intern Med 2009 169 1677 1683 10.1001/archinternmed.2009.297 19822824

[B57] White KP Driscoll MS Rothe MJ Grant-Kels JM Severe adverse cardiovascular effects of pulse steroid therapy: is continuous cardiac monitoring necessary? J Am Acad Dermatol 1994 30 768 773 10.1016/S0190-9622(08)81508-3 8176017

[B58] Moghadam-Kia S Werth VP Prevention and treatment of systemic glucocorticoid side effects Int J Dermatol 2010 49 239 248 10.1111/j.1365-4632.2009.04322.x 20465658 PMC2872100

[B59] Leong KH Koh ET Feng PH Boey ML Lipid profiles in patients with systemic lupus erythematosus J Rheumatol 1994 21 1264 1267 7966068

[B60] Petri M Spence D Bone LR Hochberg MC Coronary artery disease risk factors in the Johns Hopkins Lupus Cohort: prevalence, recognition by patients, and preventive practices Medicine (Baltimore) 1992 71 291 302 1522805 10.1097/00005792-199209000-00004

[B61] Svenson KL Lithell H Hällgren R Vessby B Serum lipoprotein in active rheumatoid arthritis and other chronic inflammatory arthritides. II. Effects of anti-inflammatory and disease-modifying drug treatment Arch Intern Med 1987 147 1917 1920 10.1001/archinte.1987.00370110045006 3675092

[B62] Choi HK Seeger JD Glucocorticoid use and serum lipid levels in US adults: the Third National Health and Nutrition Examination Survey Arthritis Rheum 2005 53 528 535 10.1002/art.21329 16082633

[B63] Miller ML Glucocorticoid-induced myopathy UpToDate 2013 2013 [http://www.uptodate.com/contents/glucocorticoid-induced-myopathy?topicKey=RHEUM%2F5171&elapsedTimeMs=3&source=see_link&view=print&displayedView=full]

[B64] Bowyer SL LaMothe MP Hollister JR Steroid myopathy: incidence and detection in a population with asthma J Allergy Clin Immunol 1985 76 234 242 10.1016/0091-6749(85)90708-0 4019954

[B65] LaPier TK Glucocorticoid-induced muscle atrophy. The role of exercise in treatment and prevention J Cardiopulm Rehabil 1997 17 76 84 10.1097/00008483-199703000-00002 9101384

[B66] Latronico N Shehu I Seghelini E Neuromuscular sequelae of critical illness Curr Opin Crit Care 2005 11 381 390 10.1097/01.ccx.0000168530.30702.3e 16015120

[B67] Wolkowitz OM Burke H Epel ES Reus VI Glucocorticoids. Mood, memory, and mechanisms Ann N Y Acad Sci 2009 1179 19 40 10.1111/j.1749-6632.2009.04980.x 19906230

[B68] Warrington TP Bostwick JM Psychiatric adverse effects of corticosteroids Mayo Clin Proc 2006 81 1361 1367 10.4065/81.10.1361 17036562

[B69] Minden SL Orav J Schildkraut JJ Hypomanic reactions to ACTH and prednisone treatment for multiple sclerosis Neurology 1988 38 1631 1634 10.1212/WNL.38.10.1631 2843795

[B70] Bolanos SH Khan DA Hanczyc M Bauer MS Dhanani N Brown ES Assessment of mood states in patients receiving long-term corticosteroid therapy and in controls with patient-rated and clinician-rated scales Ann Allergy Asthma Immunol 2004 92 500 505 10.1016/S1081-1206(10)61756-5 15191017

[B71] Swinburn CR Wakefield JM Newman SP Jones PW Evidence of prednisolone induced mood change (‘steroid euphoria’) in patients with chronic obstructive airways disease Br J Clin Pharmacol 1988 26 709 713 10.1111/j.1365-2125.1988.tb05309.x 3242575 PMC1386585

[B72] Turner R Elson E Sleep disorders. Steroids cause sleep disturbance BMJ 1993 306 1477 1478 8518660 10.1136/bmj.306.6890.1477-d PMC1677864

[B73] Brown ES Effects of glucocorticoids on mood, memory, and the hippocampus. Treatment and preventive therapy Ann N Y Acad Sci 2009 1179 41 55 10.1111/j.1749-6632.2009.04981.x 19906231

[B74] Keenan PA Jacobson MW Soleymani RM Mayes MD Stress ME Yaldoo DT The effect on memory of chronic prednisone treatment in patients with systemic disease Neurology 1996 47 1396 1402 10.1212/WNL.47.6.1396 8960717

[B75] Kershner P Wang-Cheng R Psychiatric side effects of steroid therapy Psychosomatics 1989 30 135 139 10.1016/S0033-3182(89)72293-3 2652177

[B76] Chau SY Mok CC Factors predictive of corticosteroid psychosis in patients with systemic lupus erythematosus Neurology 2003 61 104 107 10.1212/WNL.61.1.104 12847167

[B77] Goggans FC Weisberg LJ Koran LM Lithium prophylaxis of prednisone psychosis: a case report J Clin Psychiatry 1983 44 111 112 6403514

[B78] Stuck AE Minder CE Frey FJ Risk of infectious complications in patients taking glucocorticosteroids Rev Infect Dis 1989 11 954 963 10.1093/clinids/11.6.954 2690289

[B79] Saag KG Short-term and long-term safety of glucocorticoids in rheumatoid arthritis Bull NYU Hosp Jt Dis 2012 70 Suppl 1 21 25 23259654

[B80] Grijalva CG Chen L Delzell E Baddley JW Beukelman T Winthrop KL Griffin MR Herrinton LJ Liu L Ouellet-Hellstrom R Patkar NM Solomon DH Lewis JD Xie F Saag KG Curtis JR Initiation of tumor necrosis factor-α antagonists and the risk of hospitalization for infection in patients with autoimmune diseases JAMA 2011 306 2331 2339 10.1001/jama.2011.1692 22056398 PMC3428224

[B81] Allen DB Mullen M Mullen B A meta-analysis of the effect of oral and inhaled corticosteroids on growth J Allergy Clin Immunol 1994 93 967 976 10.1016/S0091-6749(94)70043-5 8006318

[B82] Allen DB Growth suppression by glucocorticoid therapy Endocrinol Metab Clin North Am 1996 25 699 717 10.1016/S0889-8529(05)70348-0 8879994

[B83] Lettgen B Jeken C Reiners C Influence of steroid medication on bone mineral density in children with nephrotic syndrome Pediatr Nephrol 1994 8 667 670 10.1007/BF00869084 7696102

[B84] Falcini F Taccetti G Trapani S Tafi L Volpi M Growth retardation in juvenile chronic arthritis patients treated with steroids Clin Exp Rheumatol 1991 9 37 40 1711944

[B85] Markowitz J Grancher K Rosa J Aiges H Daum F Growth failure in pediatric inflammatory bowel disease J Pediatr Gastroenterol Nutr 1993 16 373 380 10.1097/00005176-199305000-00005 8315544

[B86] Lai HC FitzSimmons SC Allen DB Kosorok MR Rosenstein BJ Campbell PW Farrell PM Risk of persistent growth impairment after alternate-day prednisone treatment in children with cystic fibrosis N Engl J Med 2000 342 851 859 10.1056/NEJM200003233421204 10727589

[B87] Miller W Achermann J Frankland AW Sperling M The adrenal cortex and its disorders Pediatric Endocrinology 2008 3 Philadelphia: Saunders 444 511

[B88] Canadian Pediatric Society Canadian Paediatric Surveillance Program (CPSP): 2010 results 2010 CPS; PHAC [http://www.cpsp.cps.ca/uploads/publications/Results-2010.pdf], Accessed March 5, 2013

[B89] Canadian Pediatric Society Canadian Paediatric Surveillance Program (CPSP): 2011 results 2011 CPS; PHAC [http://www.cpsp.cps.ca/uploads/publications/Results-2011.pdf], Accessed March 5, 2013

[B90] Canadian Pediatric Society Canadian Paediatric Surveillance Program (CPSP): 2012 results 2012 CPS; PHAC [http://www.cpsp.cps.ca/uploads/publications/Results-2012.pdf], Accessed May 14, 2013

[B91] Ahmet A Kim H Spier S Adrenal suppression: A practical guide to the screening and management of this under-recognized complication of inhaled corticosteroid therapy Allergy Asthma Clin Immunol 2011 7 13 10.1186/1710-1492-7-13 21867553 PMC3177893

[B92] Rix M Birkebaek NH Rosthoj S Clausen N Clinical impact of corticosteroid-induced adrenal suppression during treatment for acute lymphoblastic leukemia in children: a prospective observational study using the low-dose adrenocorticotropin test J Pediatr 2005 147 645 650 10.1016/j.jpeds.2005.06.006 16291357

[B93] Gordijn MS Gemke RJ van Dalen EC Rotteveel J Kaspers GJ Hypothalamic-pituitary-adrenal (HPA) axis suppression after treatment with glucocorticoid therapy for childhood acute lymphoblastic leukaemia Cochrane Database Syst Rev 2012 5 CD008727 10.1002/14651858.CD008727.pub2 22592733

[B94] Wood JB Frankland AW James VH Landon J A rapid test of adrenocortical function Lancet 1965 1 243 245 14238068 10.1016/s0140-6736(65)91526-6

[B95] Plager JE Cushman P Jr Suppression of the pituitary-ACTH response in man by administration of ACTH or cortisol J Clin Endocrinol Metab 1962 22 147 154 10.1210/jcem-22-2-147 14487062

[B96] Axelrod L Glucocorticoid therapy Medicine (Baltimore) 1976 55 39 65 10.1097/00005792-197601000-00003 173974

[B97] Amed S Dean H Sellers EA Panagiotopoulos C Shah BR Booth GL Laubscher TA Dannenbaum D Hadjiyannakis S Hamilton JK Risk factors for medication-induced diabetes and type 2 diabetes J Pediatr 2011 159 291 296 10.1016/j.jpeds.2011.01.017 21353243

[B98] Ho J Pacaud D Secondary diabetes in children Can J Diab 2004 28 400 405

[B99] Stratakis CA Cushing syndrome in pediatrics Endocrinol Metab Clin North Am 2012 41 793 803 10.1016/j.ecl.2012.08.002 23099271 PMC3594781

[B100] Semeao EJ Jawad AF Stouffer NO Zemel BS Piccoli DA Stallings VA Risk factors for low bone mineral density in children and young adults with Crohn’s disease J Pediatr 1999 135 593 600 10.1016/S0022-3476(99)70058-2 10547248

[B101] Boot AM Bouquet J Krenning EP de Muinck Keizer-Schrama SMPF Bone mineral density and nutritional status in children with chronic inflammatory bowel disease Gut 1998 42 188 194 10.1136/gut.42.2.188 9536942 PMC1726993

[B102] Kotaniemi A Savolainen A Kautiainen H Kröger H Estimation of central osteopenia in children with chronic polyarthritis treated with glucocorticoids Pediatrics 1993 91 1127 1130 8502514

[B103] Bhudhikanok GS Wang M-C Marcus R Harkins A Moss RB Bachrach LK Bone acquisition and loss in children and adults with cystic fibrosis: a longitudinal study J Pediatr 1998 133 18 27 10.1016/S0022-3476(98)70172-6 9672505

[B104] Conway SP Morton AM Oldroyd B Truscott JG White H Smith AH Haigh I Osteoporosis and osteopenia in adults and adolescents with cystic fibrosis: prevalence and associated factors Thorax 2000 55 798 804 10.1136/thorax.55.9.798 10950902 PMC1745849

[B105] Bardare M Bianchi ML Furia M Gandolini GG Cohen E Montesano A Bone mineral metabolism in juvenile chronic arthritis: the influence of steroids Clin Exp Rheumatol 1991 9 Suppl 6 29 31 2060174

[B106] Fantini F Beltrametti P Gallazzi M Gattinara M Gerloni V Murelli M Parrini M Evaluation by dual-photon absorptiometry of bone mineral loss in rheumatic children on long-term treatment with corticosteroids Clin Exp Rheumatol 1991 9 Suppl 6 21 28 2060173

[B107] Perez MD Abrams SA Loddeke L Shypailo R Ellis KJ Effects of rheumatic disease and corticosteroid treatment on calcium metabolism and bone density in children assessed one year after diagnosis, using stable isotopes and dual energy X-ray absorptiometry J Rheumatol 2000 27 Suppl 58 38 43 10782855

[B108] van Staa TP Cooper C Leufken HGM Bishop N Children and the risk of fractures caused by oral corticosteroids J Bone Miner Res 2003 18 913 918 10.1359/jbmr.2003.18.5.913 12733732

[B109] Halton J Gaboury I Grant R Alos N Cummings EA Matzinger M Shenouda N Lentle B Abish S Atkinson S Cairney E Dix D Israels S Stephure D Wilson B Hay J Moher D Rauch F Siminoski K Ward LM Canadian STOPP Consortium Advanced vertebral fracture among newly diagnosed children with acute lymphoblastic leukemia: results of the Canadian Steroid-Associated Osteoporosis in the Pediatric Population (STOPP) research program J Bone Miner Res 2009 24 1326 1334 10.1359/jbmr.090202 19210218 PMC3890351

[B110] Huber AM Gaboury I Cabral DA Lang B Ni A Stephure D Taback S Dent P Ellsworth J LeBlanc C Saint-Cyr C Scuccimarri R Hay J Lentle B Matzinger M Shenouda N Moher D Rauch F Siminoski K Ward LM Canadian Steroid-Associated Osteoporosis in the Pediatric Population (STOPP) Consortium Prevalent vertebral fractures among children initiating glucocorticoid therapy for the treatment of rheumatic disorders Arthritis Care Res (Hoboken) 2010 62 516 526 10.1002/acr.20171 20391507 PMC3958950

[B111] Rodd C Lang B Ramsay T Alos N Huber AM Cabral DA Scuccimarri R Miettunen PM Roth J Atkinson SA Couch R Cummings EA Dent PB Ellsworth J Hay J Houghton K Jurencak R Larché M LeBlanc C Oen K Saint-Cyr C Stein R Stephure D Taback S Lentle B Matzinger M Shenouda N Moher D Rauch F Siminoski K Ward LM Canadian Steroid-Associated Osteoporosis in the Pediatric Population (STOPP) Consortium Incident vertebral fractures among children with rheumatic disorders 12 months after glucocorticoid initiation: A national observational study Arthritis Care Res (Hoboken) 2012 64 122 131 10.1002/acr.20589 22213727 PMC4459856

[B112] Feber J Gaboury I Ni A Alos N Arora S Bell L Blydt-Hansen T Clarson C Filler G Hay J Hebert D Lentle B Matzinger M Midgley J Moher D Pinsk M Rauch F Rodd C Shenouda N Siminoski K Ward LM Canadian STOPP Consortium Skeletal findings in children recently initiating glucocorticoids for the treatment of nephrotic syndrome Osteoporos Int 2012 23 751 760 10.1007/s00198-011-1621-2 21494860 PMC4000256

[B113] Papaioannou A Morin S Cheung AM Atkinson S Brown JP Feldman S Hanley DA Hodsman A Jamal SA Kaiser SM Kvern B Siminoski K Leslie WD Scientific Advisory Council of Osteoporosis Canada 2010 clinical practice guidelines for the diagnosis and management of osteoporosis in Canada: summary CMAJ 2010 182 1864 1873 10.1503/cmaj.100771 20940232 PMC2988535

[B114] Rodrigues Pereira RM Carvalho JF Paula AP Zerbini C Domiciano DS Gonçalves H Danowski JS Marques Neto JF Mendonça LM Bezerra MC Terreri MT Imamura M Weingrill P Plapler PG Radominski S Tourinho T Szejnfeld VL Andrada NC Committee for Osteoporosis and Bone Metabolic Disorders of the Brazilian Society of Rheumatology Guidelines for the prevention and treatment of glucocorticoid-induced osteoporosis Rev Bras Reumatol 2012 52 580 593 10.1590/S0482-50042012000400009 22885424

[B115] Grossman JM Gordon R Ranganath VK Deal C Caplan L Chen W Curtis JR Furst DE McMahon M Patkar NM Volkmann E Saag KG American College of Rheumatology 2010 recommendations for the prevention and treatment of glucocorticoid-induced osteoporosis Arthritis Care Res (Hoboken) 2010 62 1515 1526 10.1002/acr.20295 20662044

[B116] National Osteoporosis Guideline Group Osteoporosis: Clinical guideline for prevention and treatment. Updated July, 2010 2010 National Osteoporosis Guideline Group [http://www.shef.ac.uk/NOGG/NOGG_Executive_Summary.pdf], Accessed March 8, 2013

[B117] National Osteoporosis Foundation Clinician’s Guide to Prevention and Treatment of Osteoporosis 2013 Washington, DC: National Osteoporosis Foundation [http://www.nof.org/files/nof/public/content/file/917/upload/481.pdf], Accessed March 8, 2013

[B118] Kanis JA Johansson H Oden A McCloskey EV Guidance for the adjustment of FRAX according to the dose of glucocorticoids Osteoporos Int 2011 22 809 816 10.1007/s00198-010-1524-7 21229233

[B119] Mushtaq T Ahmed SF The impact of corticosteroids on growth and bone health Arch Dis Child 2002 87 93 96 10.1136/adc.87.2.93 12138051 PMC1719206

[B120] Carella MJ Srivastava LS Gossain VV Rovner DR Hypothalamic-pituitary-adrenal function one week after a short burst of steroid therapy J Clin Endocrinol Metab 1993 76 1188 1191 10.1210/jc.76.5.1188 8388401

[B121] Erturk E Jaffe CA Barkan AL Evaluation of the integrity of the hypothalamic-pituitary-adrenal axis by insulin hypoglycemia test J Clin Endocrinol Metab 1998 83 2350 2354 10.1210/jc.83.7.2350 9661607

[B122] Tordjman K Jaffe A Grazas N Apter C Stern N The role of the low dose (1 microgram) adrenocorticotropin test in the evaluation of patients with pituitary diseases J Clin Endocrinol Metab 1995 80 1301 1305 10.1210/jc.80.4.1301 7714104

[B123] Tordjman K Jaffe A Trostanetsky Y Greenman Y Limor R Stern N Low-dose (1 microgram) adrenocorticotropin (ACTH) stimulation as a screening test for impaired hypothalamo-pituitary-adrenal axis function: sensitivity, specificity and accuracy in comparison with the high-dose (250 microgram) test Clin Endocrinol (Oxf) 2000 52 633 640 10.1046/j.1365-2265.2000.00984.x 10792344

[B124] Kazlauskaite R Evans AT Villabona CV Abdu TA Ambrosi B Atkinson AB Choi CH Clayton RN Courtney CH Gonc EN Maghnie M Rose SR Soule SG Tordjman K Consortium for Evaluation of Corticotropin Test in Hypothalamic-Pituitary Adrenal Insufficiency Corticotropin tests for hypothalamic-pituitary-adrenal insufficiency: a meta-analysis J Clin Endocrinol Metab 2008 93 4245 4253 10.1210/jc.2008-0710 18697868

[B125] Anderson TJ Grégoire J Hegele RA Couture P Mancini GB McPherson R Francis GA Poirier P Lau DC Grover S Genest J Jr Carpentier AC Dufour R Gupta M Ward R Leiter LA Lonn E Ng DS Pearson GJ Yates GM Stone JA Ur E 2012 update of the Canadian cardiovascular society guidelines for the diagnosis and treatment of dyslipidemia for the prevention of cardiovascular disease in the adult Can J Cardiol 2013 29 151 167 10.1016/j.cjca.2012.11.032 23351925

[B126] Davidson J Wilkinson AH Dantal J Dotta F Haller H Hernández D Kasiske BL Kiberd B Krentz A Legendre C Marchetti P Markell M van der Woude FJ Wheeler DC International Expert Panel New-onset diabetes after transplantation: 2003 International Consensus Guidelines. Proceedings of an international expert panel meeting. Barcelona, Spain, 19 February 2003 Transplantation 2003 7 SS3 SS24 12775942 10.1097/01.TP.0000069952.49242.3E

[B127] Humbert M Beasley R Ayres J Slavin R Hébert J Bousquet J Beeh KM Ramos S Canonica GW Hedgecock S Fox H Blogg M Surrey K Benefits of omalizumab as add-on therapy in patients with severe persistent asthma who are inadequately controlled despite best available therapy (GINA 2002 step 4 treatment): INNOVATE Allergy 2005 60 309 316 10.1111/j.1398-9995.2004.00772.x 15679715

[B128] Lougheed MD Lemiere C Ducharme FM Licskai C Dell SD Rowe BH Fitzgerald M Leigh R Watson W Boulet LP Canadian Thoracic Society Asthma Clinical Assembly Canadian Thoracic Society 2012 guideline update: Diagnosis and management of asthma in preschoolers, children and adults Can Respir J 2012 19 127 164 22536582 10.1155/2012/635624 PMC3373283

[B129] Devogelaer JP Goemaere S Boonen S Body JJ Kaufman JM Reginster JY Rozenberg S Boutsen Y Evidence-based guidelines for the prevention and treatment of glucocorticoid-induced osteoporosis: a consensus document of the Belgian Bone Club Osteoporos Int 2006 17 8 19 10.1007/s00198-005-2032-z 16217586

[B130] Nawata H Soen S Takayanagi R Tanaka I Takaoka K Fukunaga M Matsumoto T Suzuki Y Tanaka H Fujiwara S Miki T Sagawa A Nishizawa Y Seino Y Subcommittee to Study Diagnostic Criteria for Glucocorticoid-Induced Osteoporosis Guidelines on the management and treatment of glucocorticoid-induced osteoporosis of the Japanese Society for Bone and Mineral Research (2004) J Bone Miner Metab 2005 23 105 109 10.1007/s00774-004-0596-x 15750687

[B131] National Osteoporosis Society & Royal College of Physicians Guidelines Working Group for Bone and Tooth Society Glucocorticoid-induced osteoporosis: guidelines for prevention and treatment 2002 London: Royal College of Physicians

[B132] Homik J Cranney A Shea B Tugwell P Wells G Adachi R Suarez-Almazor M Bisphosphonates for steroid induced osteoporosis Cochrane Database Syst Rev 2000 2 CD001347 10796432 10.1002/14651858.CD001347

[B133] Adachi JD Bensen WG Brown J Hanley D Hodsman A Josse R Kendler DL Lentle B Olszynski W Ste-Marie LG Tenenhouse A Chines AA Intermittent etidronate therapy to prevent corticosteroid-induced osteoporosis N Engl J Med 1997 337 382 387 10.1056/NEJM199708073370603 9241127

[B134] Saag KG Emkey R Schnitzer TJ Brown JP Hawkins F Goemaere S Thamsborg G Liberman UA Delmas PD Malice MP Czachur M Daifotis AG Alendronate for the prevention and treatment of glucocorticoid-induced osteoporosis N Engl J Med 1998 339 292 299 10.1056/NEJM199807303390502 9682041

[B135] Cohen S Levy RM Keller M Boling E Emkey RD Greenwald M Zizic TM Wallach S Sewell KL Lukert BP Axelrod DW Chines AA Risedronate therapy prevents corticosteroid-induced bone loss: a twelve-month, multicenter, randomized, double-blind, placebo-controlled, parallel-group study Arthritis Rheum 1999 42 2309 2318 10.1002/1529-0131(199911)42:11<2309::AID-ANR8>3.0.CO;2-K 10555025

[B136] Wallach S Cohen S Reid DM Hughes RA Hosking DJ Laan RF Doherty SM Maricic M Rosen C Brown J Barton I Chines AA Effects of risedronate treatment on bone density and vertebral fracture in patients on corticosteroid therapy Calcif Tissue Int 2000 67 277 285 10.1007/s002230001146 11000340

[B137] Reid DM Devogelaer JP Saag K Roux C Lau CS Reginster JY Papanastasiou P Ferreira A Hartl F Fashola T Mesenbrink P Sambrook PN HORIZON investigators Zoledronic acid and risedronate in the prevention and treatment of glucocorticoid-induced osteoporosis (HORIZON): a multicentre, double-blind, double-dummy, randomised controlled trial Lancet 2009 373 1253 1263 10.1016/S0140-6736(09)60250-6 19362675

[B138] Roux C Reid DM Devogelaer JP Saag K Lau CS Reginster JY Papanastasiou P Bucci-Rechtweg C Su G Sambrook PN Post hoc analysis of a single IV infusion of zoledronic acid versus daily oral risedronate on lumbar spine bone mineral density in different subgroups with glucocorticoid-induced osteoporosis Osteoporos Int 2012 23 1083 1090 10.1007/s00198-011-1800-1 21975559

[B139] Saag KG Shane E Boonen S Marin F Donley DW Taylor KA Dalsky GP Marcus R Teriparatide or alendronate in glucocorticoid induced osteoporosis N Engl J Med 2007 357 2028 2039 10.1056/NEJMoa071408 18003959

[B140] Saag KG Zanchetta JR Devogelaer JP Adler RA Eastell R See K Krege JH Krohn K Warner MR Effects of teriparatide versus alendronate for treating glucocorticoid-induced osteoporosis: thirty-six–month results of a randomized, double-blind, controlled trial Arthritis Rheum 2009 60 3346 3355 10.1002/art.24879 19877063

[B141] Karras D Stoykov I Lems WF Langdahl BL Ljunggren Ö Barrett A Walsh JB Fahrleitner-Pammer A Rajzbaum G Jakob F Marin F Effectiveness of teriparatide in postmenopausal women with osteoporosis and glucocorticoid use: 3-year results from the EFOS study J Rheumatol 2012 39 600 609 10.3899/jrheum.110947 22247365

[B142] Cranney A Welch V Adachi JD Homik J Shea B Suarez-Almazor ME Tugwell P Wells G Calcitonin for the treatment and prevention of corticosteroid-induced osteoporosis Cochrane Database Syst Rev 2000 2 CD001983 10796457 10.1002/14651858.CD001983 PMC8409281

[B143] European Medicines Agency Calcitonin [bulletin] 2013 July 2012 [http://www.ema.europa.eu/ema/index.jsp?curl=pages/medicines/human/referrals/Calcitonin/human_referral_000319.jsp&mid=WC0b01ac0580024e99]

[B144] Ettinger B Black DM Mitlak BH Knickerbocker RK Nickelsen T Genant HK Christiansen C Delmas PD Zanchetta JR Stakkestad J Glüer CC Krueger K Cohen FJ Eckert S Ensrud KE Avioli LV Lips P Cummings SR Reduction of vertebral fracture risk in postmenopausal women with osteoporosis treated with raloxifene: results from a 3-year randomized clinical trial. Multiple Outcomes of Raloxifene Evaluation (MORE) Investigators JAMA 1999 282 637 645 10.1001/jama.282.7.637 10517716

[B145] Hofbauer LC Zeitz U Schoppet M Skalicky M Schüler C Stolina M Kostenuik PJ Erben RG Prevention of glucocorticoid-induced bone loss in mice by inhibition of RANKL Arthritis Rheum 2009 60 1427 1437 10.1002/art.24445 19404943

[B146] Dore RK Cohen SB Lane NE Palmer W Shergy W Zhou L Wang H Tsuji W Newmark R Denosumab RA Study Group Effects of denosumab on bone mineral density and bone turnover in patients with rheumatoid arthritis receiving concurrent glucocorticoids or bisphosphonates Ann Rheum Dis 2010 69 872 875 10.1136/ard.2009.112920 19734132 PMC6909937

[B147] Cummings SR San Martin J McClung MR Siris ES Eastell R Reid IR Delmas P Zoog HB Austin M Wang A Kutilek S Adami S Zanchetta J Libanati C Siddhanti S Christiansen C FREEDOM Trial Denosumab for prevention of fractures in postmenopausal women with osteoporosis N Engl J Med 2009 361 756 765 10.1056/NEJMoa0809493 19671655

[B148] Papapoulos S Chapurlat R Libanati C Brandi ML Brown JP Czerwiński E Krieg MA Man Z Mellström D Radominski SC Reginster JY Resch H Román Ivorra JA Roux C Vittinghoff E Austin M Daizadeh N Bradley MN Grauer A Cummings SR Bone HG Five years of denosumab exposure in women with postmenopausal osteoporosis: results from the first two years of the FREEDOM extension J Bone Miner Res 2012 27 694 701 10.1002/jbmr.1479 22113951 PMC3415620

[B149] Homik J Suarez-Almazor ME Shea B Cranney A Wells G Tugwell P Calcium and vitamin D for corticosteroid-induced osteoporosis Cochrane Database Syst Rev 2000 2 CD000952 10796394 10.1002/14651858.CD000952 PMC7046131

[B150] Institute of Medicine Dietary reference intakes for calcium and vitamin D 2011 Washington, DC: The National Academies Press 21796828

[B151] Holick MF Binkley NC Bischoff-Ferrari HA Gordon CM Hanley DA Heaney RP Murad MH Weaver CM Endocrine Society Evaluation, treatment, and prevention of vitamin D deficiency: an Endocrine Society clinical practice guideline J Clin Endocrinol Metab 2011 96 1911 1930 10.1210/jc.2011-0385 21646368

[B152] Ward L Tricco AC Phuong P Cranney A Barrowman N Gaboury I Rauch F Tugwell P Moher D Bisphosphonate therapy for children and adolescents with secondary osteoporosis Cochrane Database Syst Rev 2007 4 CD005324 17943849 10.1002/14651858.CD005324.pub2 PMC8884163

[B153] Bachrach LK Ward LM Clinical review 1: Bisphosphonate use in childhood osteoporosis J Clin Endocrinol Metab 2009 94 400 409 10.1210/jc.2008-1531 19033370

[B154] Sbrocchi AM Rauch F Jacob P McCormick A McMillan HJ Matzinger MA Ward LM The use of intravenous bisphosphonate therapy to treat vertebral fractures due to osteoporosis among boys with Duchenne muscular dystrophy Osteoporos Int 2012 23 2703 2711 10.1007/s00198-012-1911-3 22297733

[B155] Sbrocchi AM Forget S Laforte D Azouz EM Rodd C Zoledronic acid for the treatment of osteopenia in pediatric Crohn’s disease Pediatr Int 2010 52 754 761 10.1111/j.1442-200X.2010.03174.x 20524999

[B156] Lai KA Shen WJ Yang CY Shao CJ Hsu JT Lin RM The use of alendronate to prevent early collapse of the femoral head in patients with non-traumatic osteonecrosis. A randomized clinical study J Bone Joint Surg Am 2005 87 2155 2159 10.2106/JBJS.D.02959 16203877

[B157] Agarwala S Shah SB Ten year followup of avascular necrosis of femoral head treated with alendronate for 3 years J Arthroplasty 2011 26 1128 1134 10.1016/j.arth.2010.11.010 21256699

[B158] Chen CH Chang JK Lai KA Hou SM Chang CH Wang GJ Alendronate in the prevention of collapse of the femoral head in nontraumatic osteonecrosis: a two-year multicenter, prospective, randomized, double-blind, placebo-controlled study Arthritis Rheum 2012 64 1572 1578 10.1002/art.33498 22127729

[B159] Kotecha RS Powers N Lee SJ Murray KJ Carter T Cole C Use of bisphosphonates for the treatment of osteonecrosis as a complication of therapy for childhood acute lymphoblastic leukaemia (ALL) Pediatr Blood Cancer 2010 54 934 940 20127847 10.1002/pbc.22428

[B160] Leblicq C Laverdière C Décarie JC Delisle JF Isler MH Moghrabi A Chabot G Alos N Effectiveness of pamidronate as treatment of symptomatic osteonecrosis occurring in children treated for acute lymphoblastic leukemia Pediatr Blood Cancer 2013 60 741 747 10.1002/pbc.24313 23002054

[B161] Coursin DB Wood KE Corticosteroid supplementation for adrenal insufficiency JAMA 2002 287 236 240 10.1001/jama.287.2.236 11779267

[B162] Salem M Tainsh RE Jr Bromberg J Loriaux DL Chernow B Perioperative glucocorticoid coverage. A reassessment 42 years after emergence of a problem Ann Surg 1994 219 416 425 10.1097/00000658-199404000-00013 8161268 PMC1243159

[B163] Ahmed SF Tucker P Mushtaq T Wallace AM Williams DM Hughes IA Short-term effects on linear growth and bone turnover in children randomized to receive prednisolone or dexamethasone Clin Endocrinol (Oxf) 2002 57 185 191 10.1046/j.1365-2265.2002.01580.x 12153596

[B164] Allen DB Julius JR Breen TJ Treatment of glucocorticoid-induced growth suppression with growth hormone. On behalf of the National Cooperative Growth Study J Clin Endocrinol Metab 1998 83 2824 2829 10.1210/jc.83.8.2824 9709954

